# A Binary-Based Matrix Model for *Malus* Corolla Symmetry and Its Variational Significance

**DOI:** 10.3389/fpls.2020.00416

**Published:** 2020-04-28

**Authors:** Ting Zhou, Wangxiang Zhang, Donglin Zhang, Yousry A. El-Kassaby, Junjun Fan, Hao Jiang, Guibin Wang, Fuliang Cao

**Affiliations:** ^1^College of Forestry, Nanjing Forestry University, Nanjing, China; ^2^Co-Innovation Center for Sustainable Forestry in Southern China, Nanjing Forestry University, Nanjing, China; ^3^Department of Horticulture, University of Georgia, Athens, GA, United States; ^4^Yangzhou Crabapple Horticulture Company Limited, Yangzhou, China; ^5^Department of Forest and Conservation Sciences, University of British Columbia, Vancouver, BC, Canada; ^6^College of Horticulture Jinling Institute of Technology, Nanjing, China

**Keywords:** *Malus* spp., corolla symmetry, matrix model, variation, direction, degree

## Abstract

Floral symmetry (corolla symmetry) has important biological significance in plant genetics and evolution. However, it is often multi-dimensional and difficult to quantify. Here, we constructed a multi-dimensional data matrix [X Y Z] by extracting three qualitative variables with binary properties (X: corolla regularity of interval and coplanarity; Y: petal regularity of shape and size; Z: petal local regularity of curling and wrinkle) from different dimensions of petals (overall to individual, and then to the local): all petals (corolla), individual petals, and local areas of petals. To quantitatively express the degree of *Malus* corolla symmetry, these variables were then combined with weight assignments (X: 2^2^ > Y: 2^1^ > Z: 2^0^) based on their contributions to the corolla symmetry and the algorithm rule of converting binary to decimal values, which facilitated the unification of qualitative and quantitative analyses. Our results revealed significant reduction in degrees of *Malus* corolla symmetry along the direction of local to overall. Species showed higher degree of corolla symmetry than cultivars; however, taxa with stronger corolla symmetry might not necessarily be species. These findings provide new insights into the circumscription of *Malus* controversial species. The matrix model should be reference for future evaluation of angiosperm flower symmetry (lack of corolla fusion).

## Introduction

Crabapples (*Malus* spp.) are small trees and shrubs in the rose family (Rosaceae), valued for their charming flowers, colorful small fruits (≤5 cm), and diverse growth habits. They also have an added advantage of wide environmental adaptability, facilitating their world-wide prominence as landscape and gardens focal points (Wyman, 1955; Höfer et al., 2014; Lisandru et al., 2017). Crabapples have rich germplasm resources distributed almost continuously throughout the temperate Eurasia and North America (Mratinić and Akšić, 2012). Due to various characters reflecting *Malus* morphological differences (e.g., the state of the leaf in the bud, the number of styles, color of pistils or stamens, the sepals falling off or staying, and the existence or lack of stone cells in the pulp), Rehder’s (1940) revised the species and taxonomy of *Malus* Mill using 25 worldwide species collection and based on morphological differences as well as experimental results on taxonomy, Li’s (2001) study included 26 species. In Yu’s (1974) taxonomic report on Chinese *Malus* species, a total of 22 species were recognized. It should be noted that circumscriptions of some *Malus* species could be problematic as classification criteria have not been fully established (Pereira-Lorenzo et al., 2009). After a long period of natural selection and crossbreeding, *Malus* germplasm is harboring a high level of diversity, with a steadily increasing number of varieties and cultivars in relation to their wild ancestors (Muzher et al., 2007; Ulukan, 2009; Brown, 2012; Mratinić and Akšić, 2012). It is documented that more than 700 *Malus* cultivars exist worldwide, and over 200 can be found in nurseries, with approximately 60 have known parents (Chu and Tang, 2008). Most of the documented cultivars originated from selective breeding or chance seedlings (accidental discovery), thus some of their genetic backgrounds and relationships remain unclear (Jefferson, 1970; Fiala, 1994; Joneghani, 2008; Zheng et al., 2008; Guo et al., 2009).

Symmetry is a classic characteristic of floral structures in angiosperms with high aesthetic value and important biological significance (Kalisz et al., 2006; Vamosi and Vamosi, 2010). Recently, it has become an important research topic in plant phylogenetics, evolution, ecology, and molecular biology (Endress, 2001; Hileman et al., 2003; Sargent, 2004; Li and Tang, 2012; Savriama, 2018). Although the term floral symmetry refers to the entire structure with all its constitutive parts (sepals, petals, androecium, and gynoecium), the descriptions apply primarily to the perianth (particularly the corolla) (Fambrini and Pugliesi, 2016). Plant corollas display extremely high variation in size, color, structure, and function, which are in continuous remodeling to adapt to different environmental conditions and pollinators (Ma et al., 2017), thus the important foundations for corolla symmetry is constantly changing (Marazzi and Endress, 2008; Lazaro and Totland, 2014; Moyroud and Glover, 2017; De Craene, 2018). Most plants morphological studies on corolla symmetry have been restricted to the perspective of planar projection are qualitative descriptions of its evolutionary trends: radial symmetry to bilateral symmetry, and then to asymmetry, based on the number of symmetry axes (Citerne et al., 2010). Moreover, analyses have been mainly confined to taxonomic position at the species level and above (Stebbings, 1994; Gardner et al., 2016; Reyes et al., 2016). On the basis of statistical principles, few morphological studies were carried out that combine the three-dimensional spatial structures of flowers at the taxonomic level of species and below. Corolla symmetry in plants is a multi-dimensional complex (Leppik, 1972; Savriama, 2018). It is usually difficult to describe in its entirety through uni-dimensional variables. Transitions from radial symmetry to bilateral symmetry and then to asymmetry actually reflect a decrease in corolla regularity during the evolution of angiosperms. A purely qualitative description, however, not only hinders the relationship between multiple symmetry variables, but also impedes the evolutionary (variational) analysis of floral symmetry in different large populations (groups).

Binary data has the advantage of being easy to judge and obtain. Usually 1 (Yes) and 0 (No) are used to encode, so as to form one-dimensional or multi-dimensional data matrix (Cox, 1972). However, this matrix form is not easy to compare the differences between samples. The conversion from binary data to decimal data makes the matrix numerical, thus making it possible to compare differences among samples (Yao, 2006). In this study, we proposed a multi-dimensional expression concept of regularity, and extracted three qualitative variables with binary properties (X: corolla regularity; Y: petal regularity; Z: petal local regularity) from different dimensions of petals: all petals (overall) to individual petals (individual), and then to local areas of petals (local), to construct a binary three-dimensional data matrix [X Y Z]. For evaluating the degrees of *Malus* corolla symmetry, weight assignments of X (2^2^) > Y (2^1^) > Z (2^0^) were given, which converted the matrix data into decimal data. The objectives were: (1) establishing a multi-dimensional, simple, and practical method for assessing the corolla symmetry in *Malus* based on statistical principles that can unify qualitative and quantitative analyses; (2) exploring the variational trends (including directions and degrees) of *Malus* corolla symmetry among species and cultivar groups, and between parents and their progeny; and (3) providing a new theoretical basis for the circumscription of *Malus* controversial species.

## Materials and Methods

### Plant Materials

A total of 140 *Malus* taxa (including 30 species and 110 cultivars) were collected from the national repository of *Malus* spp. germplasm (Yangzhou City, Jiangsu Province, China) (Table 1). All *Malus* trees were between 7 and 10 years old, which enabled them to enter the full bloom phase. Thirty individuals of each cultivar were planted in a row at 2 m apart and 3 m between rows.

**TABLE 1:** The list of *Malus* taxa collected from the national repository of *Malus* spp. germplasm (Yangzhou City, Jiangsu Province, China).

**No. species**	**No. cultivars**	**No. cultivars**	**No. cultivars**	**No. cultivars**
1. *Malus angustifolia*	31. *M.* ‘Abundance’	61. *M.* ‘Guard’	91. *M.* ‘Prairie Rose’	121. *M.* ‘Show Time’
2. *M. baccata*	32. *M.* ‘Adam’	62. *M.* ‘Harvest Gold’	92. *M.* ‘Prairifire’	122. *M.* ‘Snowdrift’
3. *M. domestica* var. *binzi*	33. *M.* ‘Adirondack’	63. *M.* ‘Hillier’	93. *M.* ‘Professor Sprenger’	123. *M.* ‘Sparkler’
4. *M. floribunda*	34. *M.* ‘Almey’	64. *M.* ‘Hopa’	94. *M.* ‘Profusion’	124. *M.* ‘Spring Glory’
5. *M. fusca*	35. *M.* ‘Ballet’	65. *M.* ‘Indian Magic’	95. *M.* ‘Purple Gem’	125. *M.* ‘Spring Sensation’
6. *M. halliana*	36. *M.* ‘Ballet Red’	66. *M.* ‘Indian Summer’	96. *M.* ‘Purple Pendula’	126. *M.* ‘Spring Snow’
7. *M. hupehensis*	37. *M.* ‘Black Jade’	67. *M.* ‘Irene’	97. *M.* ‘Purple Prince’	127. *M.* ‘Strawberry Jelly’
8. *M. ioensis*	38. *M.* ‘Brandywine’	68. *M.* ‘John Downie’	98. *M.* ‘PurpleSpring’	128. *M.* ‘Sugar Tyme’
9. *M. kirghisorum*	39. *M.* ‘Bride’	69. *M.* ‘Kelsey’	99. *M.* × *purpurea* ‘Lemoinei’	129. *M.* ‘Sweet Sugartyme’
10. *M. mandshurica*	40. *M.* ‘Butterball’	70. *M.* ‘King Arthur’	100. *M.* ‘Radiant’	130. *M.* ‘Thunderchild’
11. *M. micromalus*	41. *M.* ‘Candymint’	71. *M.* ‘Klehm’s Improved Bechtel’	101. *M.* ‘Rainbow’	131. *M.* ‘Tina’
12. *M. ombrophila*	42. *M.* ‘Cardinal’	72 *M.* ‘Lancelot’	102. *M.* ‘Red Baron’	132. *M.* ‘Van Eseltine’
13. *M. orientalis*	43. *M.* ‘Centurion’	73. *M.* ‘Lisa’	103. *M.* ‘Red Coral’	133. *M.* ‘Velvet Pillar’
14. *M. platycarpa*	44. *M.* ‘Cinderella’	74. *M.* ‘Liset’	104. *M.* ‘Red Great’	134. *M.* ‘Waxy’
15. *M. prattii*	45. *M.* ‘Coccinella’	75. *M.* ‘Lollipop’	105. *M.* ‘Red Jade’	135. *M.* ‘Weeping Madonna’
16. *M. prunifolia*	46. *M.* ‘Coralburst’	76. *M.* ‘Louisa’	106. *M.* ‘Red Jewel’	136. *M.* ‘White Cascade’
17. *M. pumila* var. *neidzwetzkyana*	47. *M.* ‘Darwin’	77. *M.* ‘Louisa Contort’	107. *M.* ‘Red Nessy’	137. *M.* ‘Winter Gold’
18. *M. rockii*	48. *M.* ‘David’	78. *M.* ‘Makamik’	108. *M.* ‘Red Sentinel’	138. *M.* ‘Winter Red’
19. *M. sargentii*	49. *M.* ‘Dolgo’	79. *M.* ‘Mary Potter’	109. *M.* ‘Red Splendor’	139. *M.* ‘Yellow Jade’
20. *M. sieboldii*	50. *M.* ‘Donald Wyman’	80. *M.* ‘May’s Delight’	110. *M.* ‘Regal’	140. *M.* × *zumi* ‘Calocarpa’
21. *M. sieversii*	51. *M.* ‘Eleyi’	81. *M.* ‘Melaleuca Bracteata’	111. *M.* ‘Robinson’	
22. *M. sieversii subsp. xinjinensis*	52. *M.* ‘Everest’	82. *M.* ‘Molten Lava’	112. *M.* ‘Roger’s Selection’	
23. *M. sikkimensis*	53. *M.* ‘Fairytail Gold’	83. *M.* ‘Neville Copeman’	113. *M.* ‘Royal Gem’	
24. *M. spectabilis*	54. *M.* ‘Fen Balei’	84. *M.* ‘Orange Dream’	114. *M.* ‘Royal Raindrop’	
25. *M. sylvestris*	55. *M.* ‘Fenhong nichang’	85. *M.* ‘Perfect Purple’	115. *M.* ‘Royalty’	
26. *M. toringoides*	56. *M.* ‘Firebird’	86. *M.* ‘Pink Double’	116. *M.* ‘Rudolph’	
27. *M. tschonoskii*	57. *M.* ‘Flame’	87. *M.* ‘Pink Double NFU’	117. *M.* ‘Selkirk’	
28. *M. turkmenorum*	58. *M.* ‘Golden Hornet’	88. *M.* ‘Pink Pillar’	118. *M.* ‘Sentinel’	
29. *M. xiaojinensis*	59. *M.* ‘Golden Raindrop’	89. *M.* ‘Pink Princess’	119. *M.* ‘Shelley’	
30. *M. yunnanensis*	60. *M.* ‘Gorgeous’	90. *M.* ‘Pink Spires’	120. *M.* ‘Show Girl’	

### Extraction of Characteristic Variables of *Malus* Corolla Symmetry and Construction of the Matrix Model

The extraction of characteristic variables of *Malus* corolla symmetry was conducted through field observation. Normally, *Malus* corolla (single flower) consisted of five petals separated at the base. Arrangement of all the petals and their morphology, such as the shape, size, curling, wrinkle, etc., had obvious effect on their corolla symmetry. Based on a thorough understanding of the symmetrical characteristics of *Malus* corolla, on average, thirty representative flowers were selected for each taxa during their full bloom phase. Three-dimensional variables reflecting corolla symmetry, corolla regularity (X), petal regularity (Y), and petal local regularity (Z), were then extracted from different dimensions of petals [corolla (overall) to individual petals (individual), and then to local areas of petals (local)] for constructing a binary three-dimensional qualitative data matrix [X Y Z]. To enhance the symmetry expression results, two sub-dimensions were established for every dimension (X_1_, X_2_; Y_1_, Y_2_; Z_1_, Z_2_) specifically as follows:

#### Corolla Regularity (X)

**FIGURE 1:**
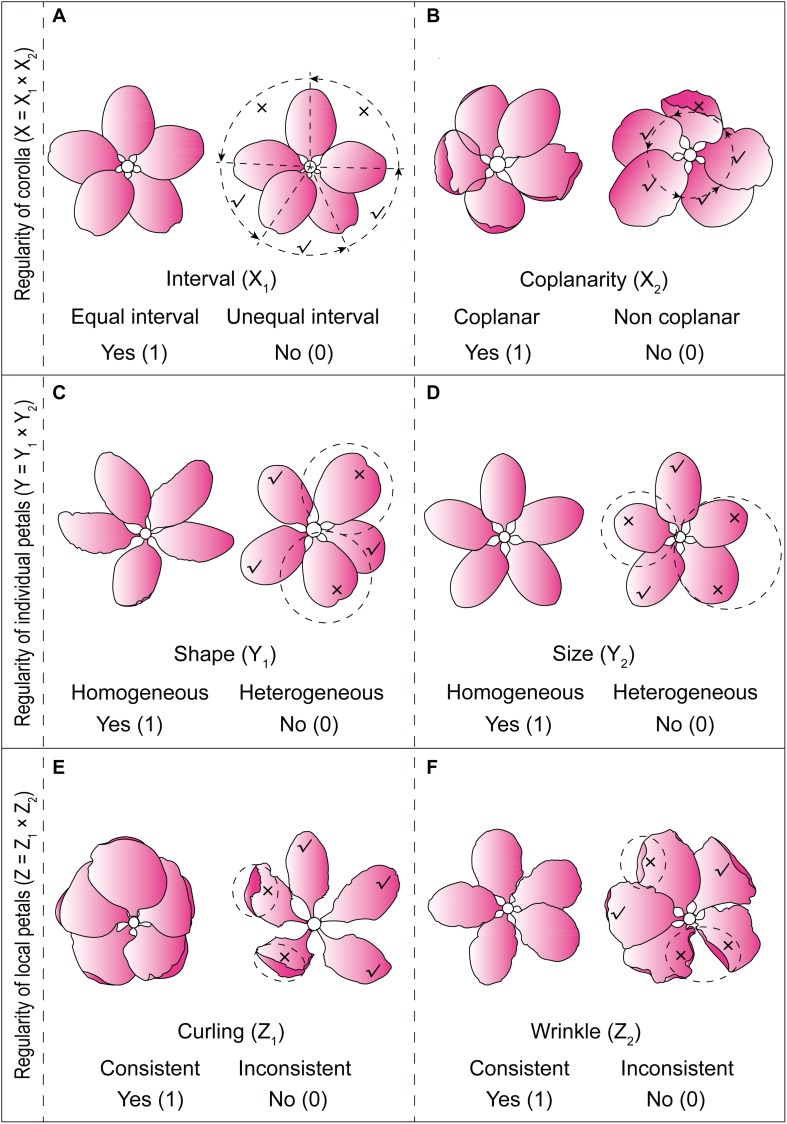
Schematic representation for determining the six sub-dimensions of *Malus* corolla symmetry. **(A)** Petal interval; **(B)** Petal coplanarity; **(C)** Petal shape homogeneity; **(D)** Petal size homogeneity; **(E)** Petal local curling consistency; **(F)** Petal local wrinkle consistency.

Sub-dimension 1 (petal interval, X_1_) refers to whether the petals on the same flower are equally spaced apart from each other on the receptacle (Figure 1A); sub-dimension 2 (petal coplanarity, X_2_) refers to whether the circumference of all petals (2/3 from the base) on the same flower are on the same geometric plane (Figure 1B).

#### Petal Regularity (Y)

Sub-dimension 1 (petal shape homogeneity, Y_1_) refers to whether the aspect ratio of all petals in the same flower and the widest position of the petal are homogeneous (Figure 1C); sub-dimension 2 (petal size homogeneity, Y_2_) refers to whether the length and width of all petals on the same flower are homogeneous (Figure 1D).

#### Petal Local Regularity (Z)

Sub-dimension 1 (petal local curling consistency, Z_1_) refers to whether the local radial extension of all petals (1/3 from the tip) in the same flower are consistent (Figure 1E); sub-dimension 2 (petal local wrinkle consistency, Z_2_) refers to whether the local lateral extension of all petals in the same flower are consistent (Figure 1F).

### Assignment of Characteristic Variables for *Malus* Corolla Symmetry and Assessment of the Degree of Symmetry

#### Assignment Rules for the Three-Dimensional Variables

Every sub-dimension is a binary trait and values were assigned based on a binary rule of 1 (Yes) and 0 (No). The final assigned value for each dimension was obtained by multiplication, i.e., X = X_1_ × X_2_; Y = Y_1_ × Y_2_; Z = Z_1_ × Z_2_. When the values of both sub-dimensions were 1 (Yes), the final value of that dimension would be 1 (Yes). Otherwise, the final value would be 0 (No).

#### Weight Coefficient Assignment Rules for the Three Variables

According to contributions to the regularity of *Malus* corolla symmetry of these three-dimensional variables, corolla (X), petal (Y), and petal local (Z) regularity, different weight coefficients were assigned to them (X > Y > Z). The assignment of weight coefficients was carried out based on positional effects, i.e., dimensional weight coefficient (*C*_j_) = 2 ^(*j*^ ^– 1)^ (where j: variable sequences in matrix [X Y Z] from right to left) (Yao, 2006).

#### Assessment of Degree of Corolla Symmetry

According to the algorithm rule of converting binary value to decimal value (Yao, 2006), the three-dimensional variable matrix (binary) was multiplied against the three-dimensional variables weight coefficient matrix (decimal) to calculate the symmetry index (SI), i.e., SI = X × 2^(3 – 1)^ + Y × 2^(2 – 1)^ + Z × 2^(1 – 1)^. This index reflects the degree of corolla symmetry, which was the greater the value, the stronger the regularity and the higher the degree of symmetry. This is illustrated as follows:

$$\left[1 1 1\right]=1\times 2^{\left(3-1\right)}+1\times 2^{\left(2-1\right)}+1\times 2^{\left(1-1\right)}$$

=7

$$\left[1 1 0\right]=1\times 2^{\left(3-1\right)}+1\times 2^{\left(2-1\right)}+0\times 2^{\left(1-1\right)}$$

=6

$$\left[1 0 1\right]=1\times 2^{\left(3-1\right)}+0\times 2^{\left(2-1\right)}+1\times 2^{\left(1-1\right)}$$

=5

$$\left[1 0 0\right]=1\times 2^{\left(3-1\right)}+1\times 2^{\left(2-1\right)}+0\times 2^{\left(1-1\right)}$$

=4

$$\left[0 1 1\right]=0\times 2^{\left(3-1\right)}+1\times 2^{\left(2-1\right)}+1\times 2^{\left(1-1\right)}$$

=3

$$\left[0 1 0\right]=0\times 2^{\left(3-1\right)}+1\times 2^{\left(2-1\right)}+0\times 2^{\left(1-1\right)}$$

=2

$$\left[0 0 1\right]=0\times 2^{\left(3-1\right)}+0\times 2^{\left(2-1\right)}+1\times 2^{\left(1-1\right)}$$

=1

$$\left[0 0 0\right]=0\times 2^{\left(3-1\right)}+0\times 2^{\left(2-1\right)}+0\times 2^{\left(1-1\right)}$$

=0

**FIGURE 2:**
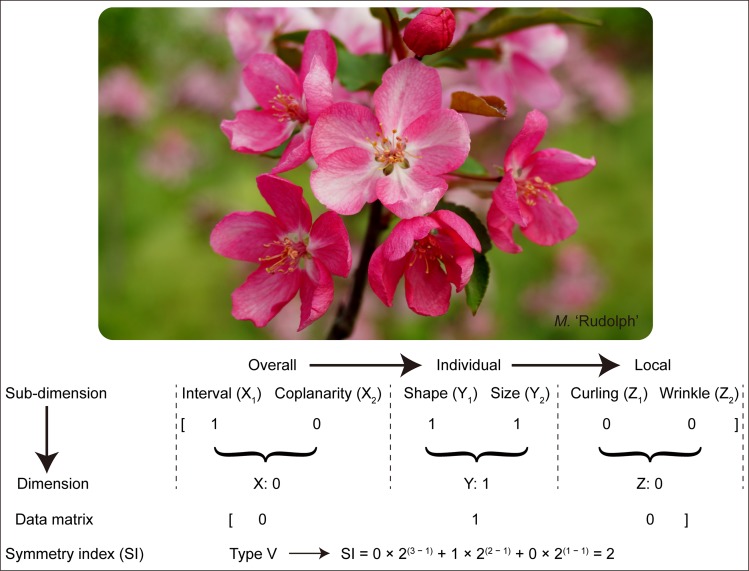
Schematic representation for the determination of corolla symmetry of *M.* ‘Rudolph.’ The original corolla symmetry data matrix was determined based on the schematic representation in Figure 1. And the final assigned value for each dimension was obtained by multiplication, i.e., X = X_1_ × X_2_; Y = Y_1_ × Y_2_; Z = Z_1_ × Z_2_. Symmetry index (SI) of *M.* ‘Rudolph’ was calculated due to the formula: SI = SI = X × 2^(3 – 1)^ + Y × 2^(2 – 1)^ + Z × 2^(1 – 1)^.

Taking the cultivar of *M.* ‘Rudolph’ as an example (Figure 2): Based on the schematic representation assignment for determining the six sub-dimensions of *Malus* corolla symmetry and the rules for binary variables, the original *Malus* corolla symmetry data matrix was [1 0 1 1 0 0]. Then the final assigned value for each dimension was obtained by multiplication, i.e., X = X_1_ × X_2_ = 0; Y = Y_1_ × Y_2_ = 1; Z = Z_1_ × Z_2_ = 0, which belonged to the symmetry type V [0 1 0] (see below). According to the algorithm rule of converting binary value to decimal value, symmetry index (SI) of *M.* ‘Rudolph’ was calculated: SI = 0 × 2^(3 – 1)^ + 1 × 2^(2 – 1)^ + 0 × 2^(1 – 1)^ = 2.

### Data Processing

Origin 9.0 and Adobe Illustrator CS5 software were used for function fitting and graph plotting.

## Results

### Classification of *Malus* Taxa Based on the Degree of Corolla Symmetry

**FIGURE 3:**
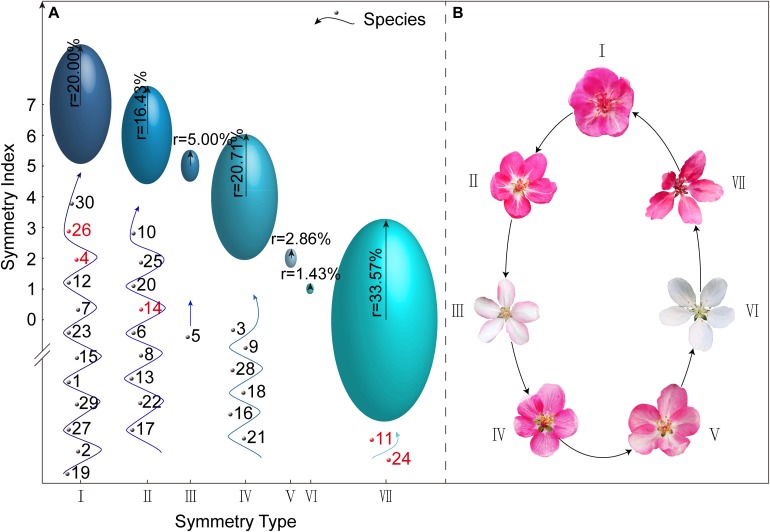
Classification of the *Malus* corolla symmetry type and the representative corolla schematic of different types. **(A)** Classification of *Malus* taxa based on the degree of corolla symmetry. The position of each ellipsoid center is the intersection of the corolla symmetry type and its corresponding symmetry index. The length of radial radius of each ellipsoid represents the percentage of *Malus* taxa included in this corolla symmetry type. The numbers at the bottom of each ellipsoid in all the symmetry types are codes of respective species, of which the red numbers represent those with controversial taxonomic status. **(B)** The representative corolla schematic of seven symmetry types.

Using the [X Y Z] three-dimensional data matrix, we classified the 140 *Malus* taxa (30 species and 110 cultivars) based on the degree of corolla symmetry. Seven types were determined: I [1 1 1], II [1 1 0], III [1 0 1], IV [1 0 0], V [0 1 0], VI [0 0 1], and VII [0 0 0], and the symmetry indices were 7, 6, 5, 4, 2, 1, and 0 points, respectively (Figure 3A). Symmetry type [0 1 1] with the index of 3 was not included in this study. Figure 3B shows the representative corolla diagrams in each type. Corolla symmetry images of all 140 *Malus* taxa were shown in Supplementary Figure S1. The distribution of *Malus* taxa among the seven types was unbalanced (with a variable coefficient of 82.46%); and they were primarily distributed in Type I, II, IV, and VII (90.71%).

### Comparative Analysis on Variational Trends of Corolla Symmetry Between *Malus* Species and Cultivars

**FIGURE 4:**
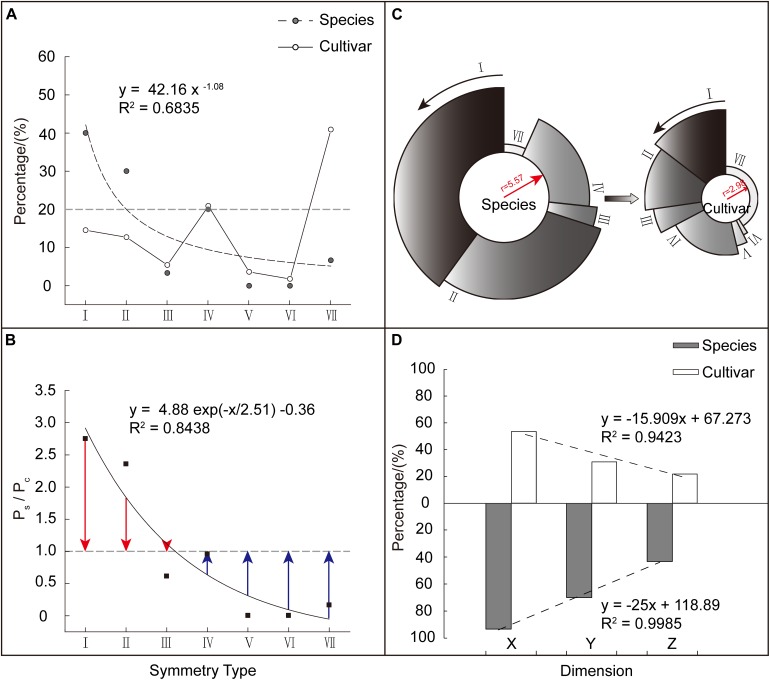
Comparison of corolla symmetry between *Malus* species and cultivars. **(A)** Composition and weight distribution of various corolla symmetry types in *Malus* species and cultivar groups. **(B)** The weighting ratio (P_s_/P_c_) distribution of the different corolla symmetry types in the two groups. Red column length (P_s_/P_c_ – 1) stands for relative weight (the weight of corolla symmetry type I, II, and III in species group exceeds that in cultivar group). Blue column length (1 – P_s_/P_c_) stands for relative weight (the weight of corolla symmetry type IV, V, VI, and VII in cultivar group exceeds that in species group). **(C)** Comparison of the degree of corolla symmetry between the two major groups. The radius of the circle represents the value of the integrated (average) symmetry index, while the percentage occupied by the circumference represents the weight occupied by each corolla symmetry type. **(D)** Distribution of regularity weights at the X, Y, and Z dimensions of the two major groups.

Based on the aforementioned seven corolla symmetry types, we divided the 140 *Malus* taxa into two groups: species (s: 30) and cultivars (c: 110). We found that among these two major groups, the distribution of the seven corolla symmetry types was extremely heterogeneous (CV_s_ = 1.12, CV_c_ = 0.95). The species group showed a significant power function distribution with a unilateral decreasing trend (*R*^2^ = 0.6835). Using a weight of 20% as the reference, taxa in species group were mainly distributed in Type I (7 points), II (6 points), and IV (4 points), which accounted for 90% of the weight distribution of all types (I to VII). The cultivar group showed a fluctuating distribution. Taxa in this group were mainly distributed in Type IV (4 points) and VII (0 points), which accounted for 61.82% of the weight distribution of all types (I to VII) (Figure 4A).

To further analyze the weight magnitude relationship in corolla symmetry types between the species and cultivars, we constructed a weight ratio (P_s_/P_c_) of different types for these two major groups. We found that P_s_/P_c_ showed a significant ExpDec1 function distribution with a unilateral decreasing trend (*R*^2^ = 0.8438). Among these symmetry types, Type I (7 points), II (6 points), and III (5 points) had P_s_/P_c_ > 1 (P_s_/P_c_ = 1 + red column length), while the other corolla symmetry types had P_s_/P_c_ < 1 (P_s_/P_c_ = 1 − blue column length) (Figure 4B). This shows that the decreased weight in the corolla symmetry types of I (7 points), II (6 points), and III (5 points), were allocated to the other four types. In addition, the lower the degree of corolla symmetry, the greater the increase in its weight (Figure 4B). This decrease/increase in weight ultimately causes a reduction in the degree of corolla symmetry in the cultivar group. As shown in Figure 4C, the species group had a higher integrated (average) symmetry index than the cultivar group, with values of 5.57 and 2.98, respectively.

Figure 4D shows the regularity weight distribution of *Malus* species and cultivar groups at the X, Y, and Z dimensions. We can see that from the X to Y, and then to Z dimension, the regularity weight distribution of the two major groups showed a decreasing trend. Furthermore, the weight differences between the two groups at the Y and Z dimensions were greater than X dimension (P_s_/P_c_’_x_ = 1.74, P_s_/P_c_’_Y_ = 2.26, and P_s_/P_c_’_z_ = 1.99). This validates the weight assignment relationship of X > Y > Z and shows that, compared with the species group, the reduction in the degree of corolla symmetry in cultivar group was mostly presented at the Y and Z dimensions, i.e., in the micro level of petals and not the macro level.

### Comparative Analysis on Variational Trends of Corolla Symmetry Between Parents and Their Offspring

**FIGURE 5:**
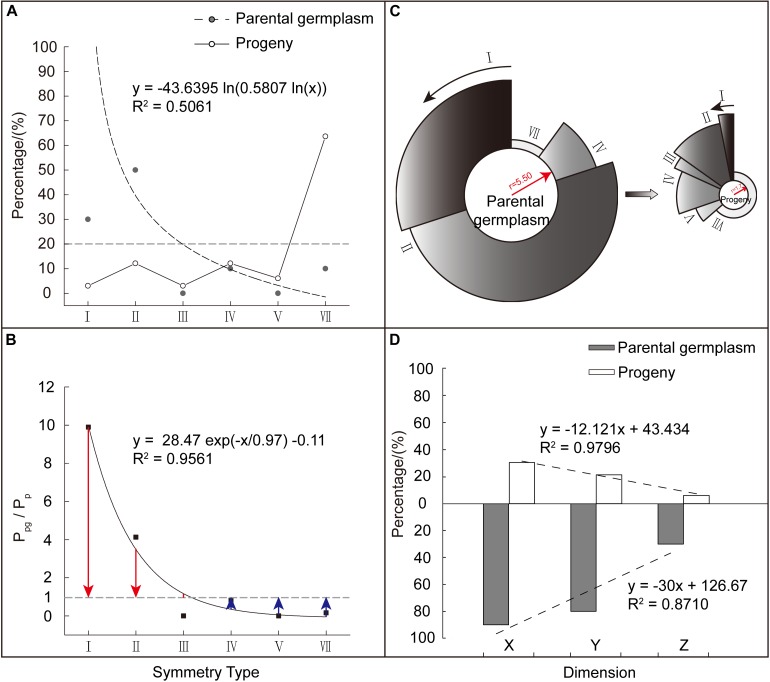
Comparison of corolla symmetry between *Malus* parental and progeny classes. **(A)** Composition and weight distribution of various corolla symmetry types in *Malus* parental and progeny classes. **(B)** The weighting ratio (P_pg_/P_p_) distribution of the different corolla symmetry types in the two classes. Red column length (P_pg_/P_p_ – 1) stands for relative weight (the weight of corolla symmetry type I, II, and III in parental class exceeds that in progeny class). Blue column length (1 – P_pg_/P_p_) stands for relative weight (the weight of corolla symmetry type IV, V, and VII in progeny class exceeds that in parental class). **(C)** Comparison of the degree of corolla symmetry between the two major classes. The radius of the circle represents the value of the integrated (average) symmetry index, while the percentage occupied by the circumference represents the weight occupied by each corolla symmetry type. **(D)** Distribution of regularity weights at the X, Y, and Z dimensions of the two major classes.

From the literature, 33 out of 110 tested *Malus* cultivars could be completely or partially traced back to their parental taxa (10 species with the degree of corolla symmetry data involved in this study) (Jefferson, 1970; Fiala, 1994; Zheng et al., 2008; Guo et al., 2009) (Table 2). To validate the variational trends of corolla symmetry from *Malus* species to cultivars, another two major classes of parents (p: 10) and progeny (pg: 33) were constructed. We found that these two major classes did not contain Type VI [0 0 1] and the distributions of the six corolla symmetry types were also extremely heterogeneous (CV_pg_ = 1.18, CV_p_ = 1.40). The parental class showed a significant logarithmic distribution with a unilateral decreasing trend (*R*^2^ = 0.5061). Using the same weight of 20% as the reference, taxa in parental class was mainly distributed in Type I (7 points) and II (6 points), which accounted for 80% of the weight distribution of all types (I to VII). The progeny class also showed a fluctuating distribution. Taxa in this group were mainly distributed in Type VII (0 points, 63.64%) (Figure 5A).

**TABLE 2:** Parental traceability and corolla symmetry type in *Malus* spp.

**No.**	**Progeny**	**Breeding lines**	**References**	**Variational trend of corolla symmetry types**	**Symmetry index of corolla symmetry types**	**Is SI of progeny ¿ maximal SI of parents?**
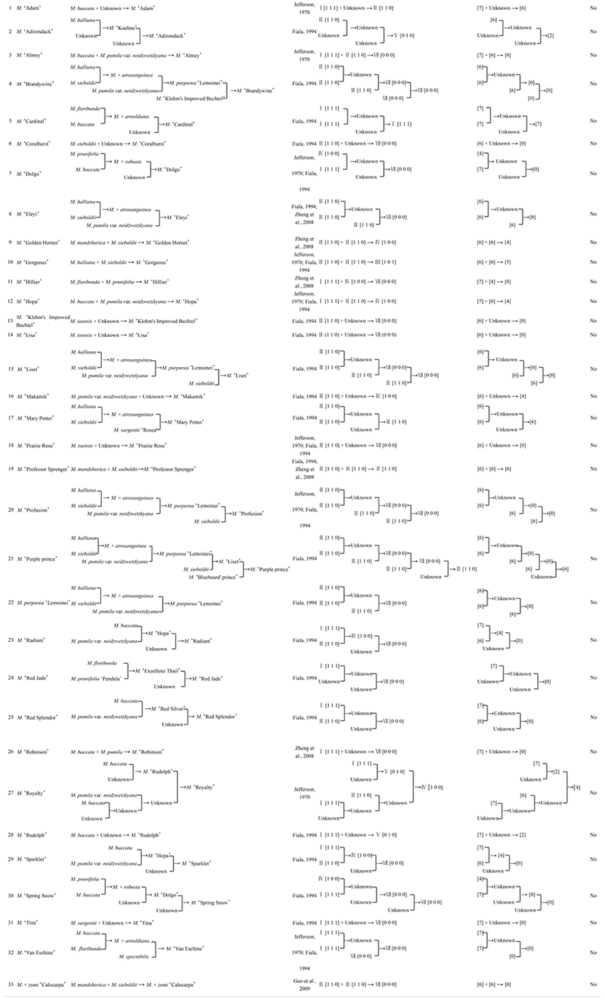						

To analyze the weight magnitude relationship in corolla symmetry types between the parental and progeny classes, the weight ratio (P_pg_/P_p_) of the different types were similarly constructed. We found that P_pg_/P_p_ showed more significant ExpDec1 function distribution with a unilateral decreasing trend (*R*^2^ = 0.9561). Among these symmetry types, Type I (7 points), II (6 points), and III (5 points) had P_pg_/P_p_ > 1 (P_pg_/P_p_ = 1 + red column length), while the other corolla symmetry types had P_pg_/P_p_ < 1 (P_pg_/P_p_ = 1 − blue column length) (Figure 5B). This indicates that the decreased weight in the corolla symmetry types of I (7 points), II (6 points), and III (5 points), were allocated to the other three types. The lower the degree of corolla symmetry, the greater the increase in its weight (Figure 5B). This decrease/increase in weight ultimately also causes a reduction in the degree of corolla symmetry in the progeny class. As shown in Figure 5C, the parental class had a higher integrated (average) symmetry index than the progeny class, with values of 5.50 and 1.70, respectively.

Figure 5D shows the regularity weight distribution of the parental and progeny classes at the X, Y, and Z dimensions. We can see that from the X to Y, and then to Z dimension, the regularity weight distribution of the two major classes also showed a decreasing trend. The weight difference between the two classes was the greatest at the Z dimension, followed by the Y and then the X dimensions (P_pg_/P_p_’_x_ = 2.97, P_pg_/P_p_’_Y_ = 3.77, and P_pg_/P_p_’_z_ = 4.95). Again this validates the weight assignment relationship of X > Y > Z and shows that, compared with the parental class, the reduction in the degree of corolla symmetry in progeny class was mostly presented at the Y and Z dimensions, i.e., in the micro level of petals and not the macro level.

The parental traceability and corolla symmetry type in *Malus* spp. were shown in Table 2. To validate the variational trend of overall reduction in the degree of corolla symmetry from *Malus* species to cultivar groups, and from parental to progeny classes more specifically, we compared the symmetry indices of the aforementioned 33 completely or locally traced cultivars (progeny) with their corresponding parents based on the breeding lines. We found that the symmetry indices in any progeny did not exceed the highest value of their parents.

## Discussion

### The Effectiveness of the Binary-Based Three-Dimensional Matrix Model in Revealing the Variation of *Malus* Corolla Symmetry

Most studies on the morphology of corolla symmetry in plants have been restricted to the perspective of planar projection, are qualitative descriptions of its evolutionary trends based on the number of symmetry axes (Citerne et al., 2010). Corolla symmetry in plants is a multi-dimensional complex (Leppik, 1972; Savriama, 2018). It is usually difficult to describe in its entirety through uni-dimensional variables. In addition, the purely qualitative description not only hinders the relationship between multiple symmetry variables, but also impedes the evolutionary (variational) analysis of floral symmetry in different large populations (groups). In this study, we proposed a multi-dimensional expression concept of regularity and extracted three characteristic variables with binary properties (X: corolla regularity of interval and coplanarity; Y: petal regularity of shape and size; Z: petal local regularity of curling and wrinkle) from different dimensions of petals: all petals (overall) to individual petals (individual), and then to local areas of petals (local), to construct a qualitative three-dimensional data matrix [X Y Z]. The weight assignment method (X: 2^2^ > Y:2^1^ > Z: 2^0^) was used to convert binary qualitative data into decimal quantitative data to obtain quantitative corolla symmetry indices for *Malus* spp. This method had numerous advantages, such as high expressivity, stability, and discrimination, and was easily measured, which effectively unified qualitative and quantitative analyses and revealed the variational trend of *Malus* corolla symmetry. From species to cultivars, the degree of corolla symmetry showed a significant decreasing trend. Species had higher corolla symmetry than cultivars ($\bar{{\mathrm{SI}}_{S\,\text{pecies}}}$ = 5.57; $\bar{{\mathrm{SI}}_{C\,\text{ultivars}}}$ = 2.98), but taxa with stronger corolla symmetry might not necessarily be species [e.g., *M*. ‘Strawberry Jelly’ (SI = 7), *M*. ‘Royal Raindrop’ (SI = 7)].

### The Reduction of *Malus* Corolla Symmetry Occurs Along the Direction of Local to Overall, Which Demonstrates the Process of Quantitative to Qualitative Changes

Corolla symmetry is one of the classic characteristics of floral structure in plants. According to the number of axes of symmetry, it can be classified into three types, namely, radial symmetry (with multiple axes of symmetry), bilateral symmetry (with one axis of symmetry), and asymmetry (without any axes of symmetry) (Rudall and Bateman, 2004; Spencer and Kim, 2017). Radial symmetry has been considered as the ancestral flower type for angiosperms, while bilateral symmetry considered to evolve several times independently from radial symmetry and asymmetry evolved from bilateral symmetry (Takhtajan, 1991; Donoghue et al., 1998; Cubas, 2004; Endress, 2011, 2012; Hileman, 2014; Zhong et al., 2017). Regrettably, these previous studies only indicated the direction of evolution but disregarded the degree because most of them were based on fossil records and without statistical evidence. In this study, we suggest that radial symmetry is a highly regular symmetry type, which requires the shape, size, and arrangement of all petals in the corolla to be strongly uniform on both sides of multiple axes of symmetry. Bilateral symmetry is a moderately regular symmetry type and requires the shape, size, and arrangement of all petals in the corolla to be strongly uniform at one axis of symmetry. Asymmetry is an irregular symmetry type. Transitions from radial symmetry to bilateral symmetry and then to the asymmetry actually reflect a decrease in corolla regularity during the evolution of angiosperms. Based on these findings, statistical analyses of the corolla regularity of groups and individuals at the X, Y, and Z dimensions from *Malus* species to cultivars were carried out. We not only found that radial symmetry (Type I) transforms into bilateral symmetry and asymmetry (Type II to VII), but also quantitatively reflected the degree in reduction of corolla symmetry (46.43%), and found that the contribution of the three-dimensional variables was X < Y < Z. The proportion of irregular taxa was 49.29, 67.86, and 77.86%, respectively. This shows that the reduction in corolla symmetry from *Malus* species to cultivars occurs along the direction of local to overall and demonstrates the process of quantitative changes to qualitative changes. It is not difficult to understand that changes in petal shape or size (Y) is an essential criterion for changes in corolla symmetry (Moyroud and Glover, 2017). Changes in petal local curling and wrinkle consistency (Z) can indirectly affect the shape and size of the planar projections of petals (Y), thereby limiting the course of axes of corolla symmetry, and decreasing their number, ultimately demoting the symmetry type. With regard to the biological significance of this variational trend, many researchers believe that bilaterally symmetrical flowers can increase the selectivity of specific pollinators, increase the accuracy of pollination, and thereby ensure the reproductive success (Neal and Anderson, 2005; Armbruster et al., 2009; Fenster et al., 2009; Ushimaru et al., 2009). In the future, the mapping relationship between corolla symmetry and fruit setting rate could be examined, which may reveal the effects of corolla symmetry on pollination in *Malus* taxa.

### The Variational Trends of *Malus* Corolla Symmetry Have Specific Reference Value for the Circumscription of Its Controversial Species

In biology, “species” is often defined as a taxon with specific morphological and biological characteristics (i.e., reproductive barriers) and with a natural distribution (Aldhebiani, 2017). Globally, there are approximately 37–60 species in the genus *Malus* (Li, 1989). However, circumscriptions of some species are still controversial as the gradual reduction in the natural distribution zones and inconsistency in the definition and taxonomic criteria for *Malus* species (den Boer, 1959). McVaugh (1943) suggested that *M. platycarpa* Rehder might be a hybrid of *M. coronaria* (L.) Mill and *M. domestica* Borkh. Li (2001) concluded that *M. floribunda* Siebold ex Van Houtte was a hybrid of *M. prunifolia* (Willd.) Borkh. and *M. sieboldii* (Regel) Rehder. Wasson (2004) considered *M. micromalus* (Makino) to be a natural hybrid of *M. baccata* (L.) Borkh and *M. spectabilis* (Sol.) Borkh., *M. zumi* (Matsum.) Rehder as the natural hybrid of *M. mandshurica* (Maxim.) Kom. ex Juz. and *M. sieboldii* (Regel) Rehder, and *M. robusta* (CarriŠre) Rehder as the hybrid of *M. baccata* (L.) Borkh and *M. prunifolia* (Willd.) Borkh. No wildtype specimens of *M. spectabilis* (Sol.) Borkh. have been observed. Shi et al. (2005) suggested that *M. toringoides* (Rehder) Hughes might be a hybrid of *M. transitoria* (Batalin) C. K. Schneid. and *M. kansuensis* (Batalin) C. K. Schneid (Table 3). While in the Manual of Cultivated Trees and Shrubs and Chinese Fruit Taxonomy, the taxa mentioned above were all defined as *Malus* species that have been well-accepted by most researchers (Rehder, 1940; Yu, 1979). In this study, we found that *M. floribunda* and *M. toringoides* both had the highest degree of corolla symmetry (Type I, regular for all three dimensions), and that *M. platycarpa* was relatively symmetrical but to a lesser extent (Type II, only the Z dimension is irregular out of the three dimensions), which completely or generally matches the corolla symmetry characteristics of *Malus* species. It would be questionable if they were not considered as species in the genus *Malus*. On the contrary, *M. micromalus* and *M. spectabilis* both had the poorest corolla symmetry (Type VII, irregular for all three dimensions). We believe that they are highly unlikely to be species in the genus *Malus*. Our results provide another method to aid in the circumscription of *Malus* controversial species, as well as some inspirations for future researchers. In addition to natural distribution zones, the variation patterns of some macro-traits [e.g., flowers (flower wholes, petals, pistils, stamens, sepals, etc.), leaves, fruits] or micro-traits (e.g., pollen exine ornamentation) may also be bases for the circumscription of species.

**TABLE 3:** Controversial *Malus* species that have been published and their possible breeding lines.

**No.**	**Controversial species**	**Breeding lines/Remarks**	**References**
1	*M. platycarpa* Rehder	*M. coronaria* (L.) Mill × *M. domestica* Borkh.	McVaugh, 1943
2	*M. floribunda* Siebold ex Van Houtte	*M. prunifolia* (Willd.) Borkh. × *M. sieboldii* (Regel) Rehder.	Li, 2001
3	*M. micromalus* (Makino)	*M. baccata* (L.) Borkh × *M. spectabilis* (Sol.) Borkh.	Wasson, 2004
4	*M. zumi* (Matsum.) Rehder	*M. mandshurica* (Maxim.) Kom. ex Juz. × *M. sieboldii* (Regel) Rehder.	
5	*M. robusta* (CarriŠre) Rehder	*M. baccata* (L.) Borkh × *M. prunifolia* (Willd.) Borkh	
6	*M. spectabilis* (Sol.) Borkh.	No wildtype specimens have been observed.	
7	*M. toringoides* (Rehder) Hughes	*M. transitoria* (Batalin) C. K. Schneid. × *M. kansuensis* (Batalin) C. K. Schneid	Shi et al., 2005

Certainly, with an eye to the stamens, pistils or the sepals of *Malus* spp. or even based on the taxonomic level of the Rosaceae family, do such variational patterns still exist in their phenotypes? Is this matrix model construction method applicable for their floral parts or is further optimization required? These questions deserve further exploration.

## Data Availability Statement

All datasets generated for this study are included in the article/Supplementary material.

## Author Contributions

TZ, WZ, and FC designed the experiments. TZ, JF, and HJ performed the experiments. TZ, WZ, JF, HJ, DZ, and FC analyzed the data. TZ and WZ wrote the manuscript. TZ, WZ, DZ, YE-K, GW, and FC revised the manuscript. All authors read and approved the final manuscript.

## Conflict of Interest

The authors declare that the research was conducted in the absence of any commercial or financial relationships that could be construed as a potential conflict of interest.
