# Supplementary material for: A Binary-Based Matrix Model for Malus Corolla Symmetry and Its Variational Significance
Source: Front Plant Sci. 2020 Apr 28;11:416. doi: 10.3389/fpls.2020.00416 (PMC7198884; doi:10.3389/fpls.2020.00416)
Supplement: Supplementary file 1 [file Image_1.pdf]

Species

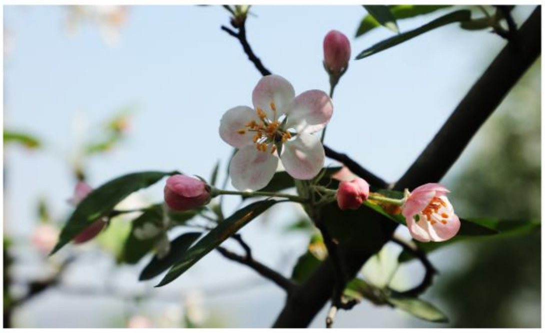

Malus angustifolia, Type Ⅰ[1 1 1 1 1 1]

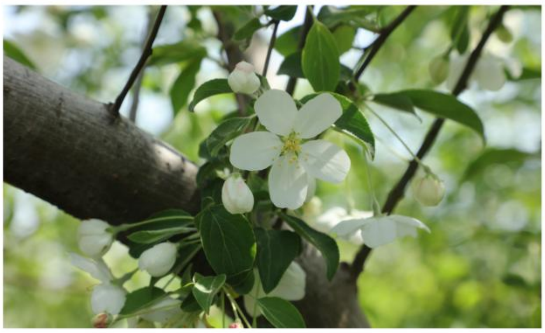

M. baccata, Type Ⅰ[1 1 1 1 1 1]

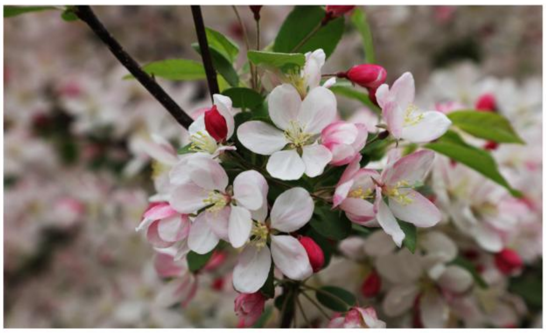

M. floribunda, Type Ⅰ[1 1 1 1 1 1]

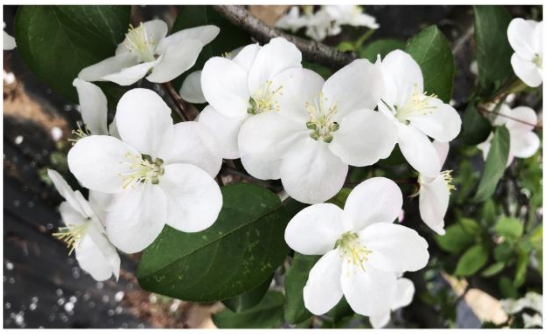

M. hupehensis, Type Ⅰ[1 1 1 1 1 1]

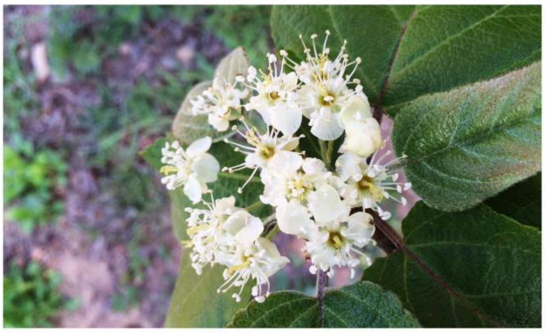

M. ombrophila, Type Ⅰ[1 1 1 1 1 1]

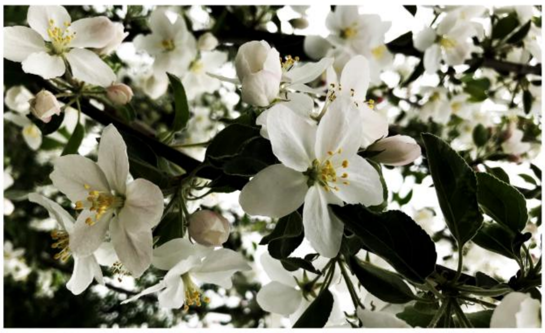

M. prattii, Type Ⅰ[1 1 1 1 1 1]

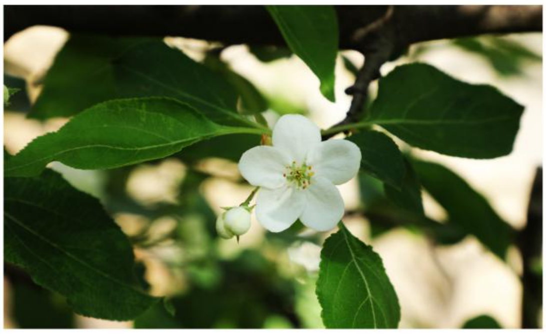

M. sargentii, Type Ⅰ[1 1 1 1 1 1]

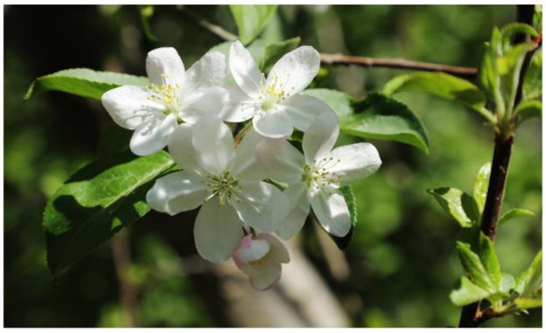

M. sikkimensis, Type Ⅰ[1 1 1 1 1 1]

Species

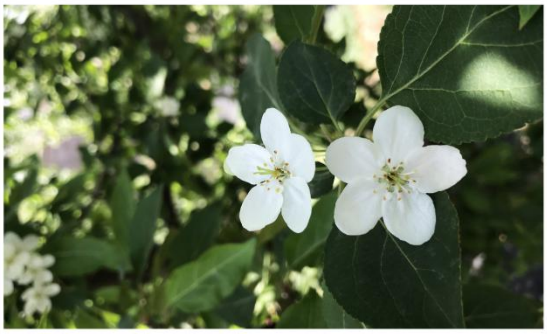

M. toringoides, Type Ⅰ[1 1 1 1 1 1]

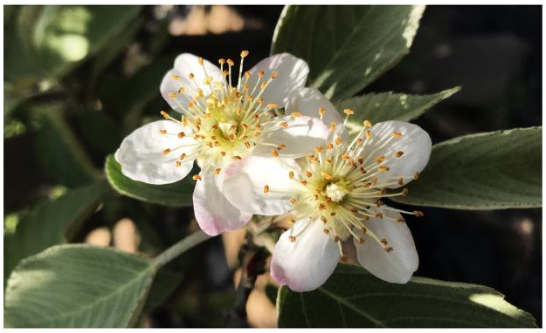

M. tschonoskii, Type Ⅰ[1 1 1 1 1 1]

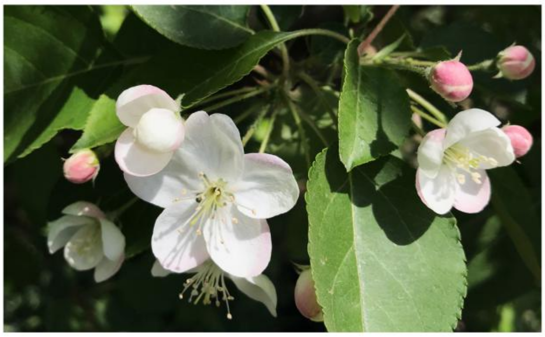

M. xiaojinensis, Type Ⅰ[1 1 1 1 1 1]

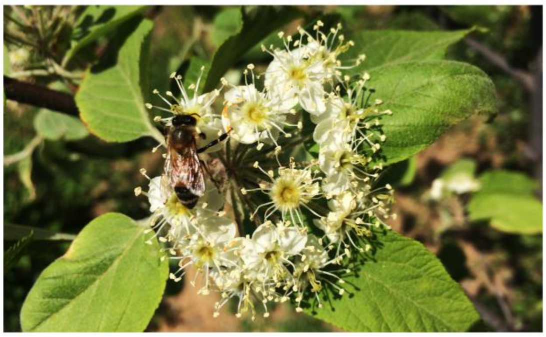

M. yunnanensis, Type Ⅰ[1 1 1 1 1 1]

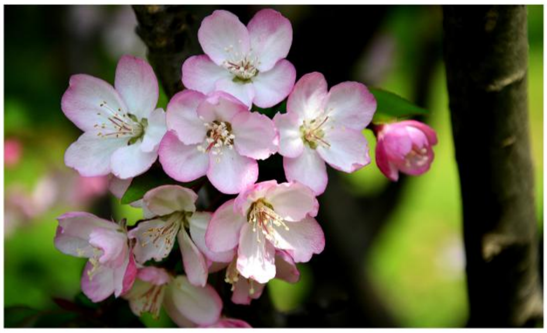

M. halliana, Type Ⅱ[1 1 1 1 1 0]

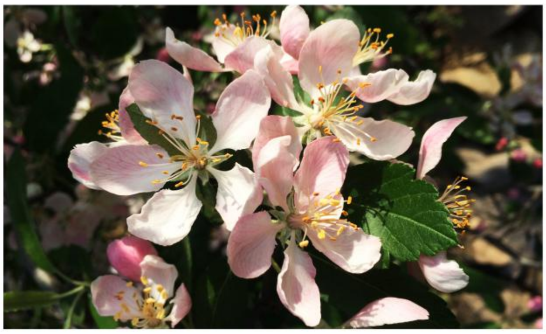

M. ioensis, Type Ⅱ[1 1 1 1 1 0]

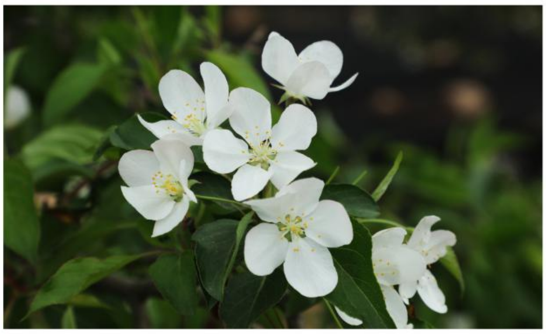

M. mandshurica, Type Ⅱ[1 1 1 1 1 0]

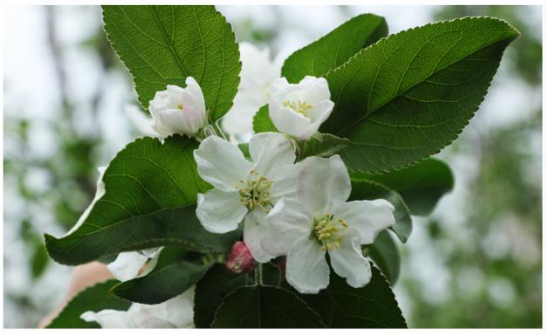

M. orientalis, Type Ⅱ[1 1 1 1 1 0]

Species

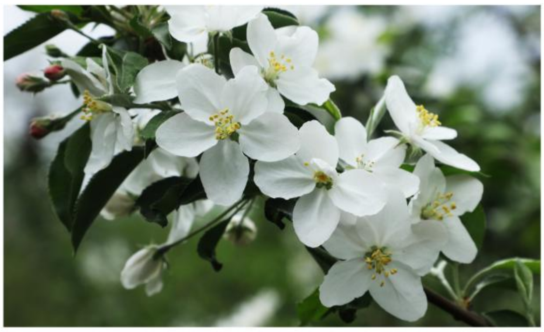

M. platycarpa, Type Ⅱ [1 1 1 1 1 0]

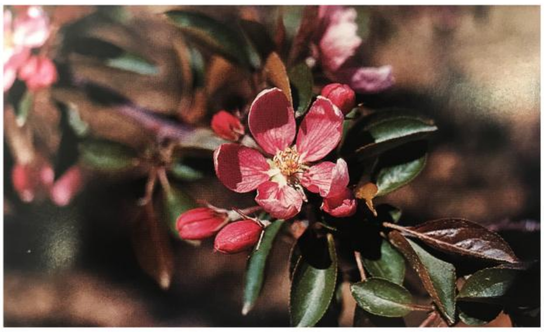

M. pumila var. *neidzwetzkyana*, Type Ⅱ [1 1 1 1 1 0]

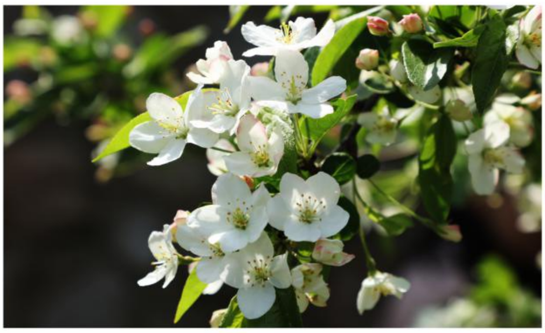

M.sieboldii, Type Ⅱ [1 1 1 1 1 0]

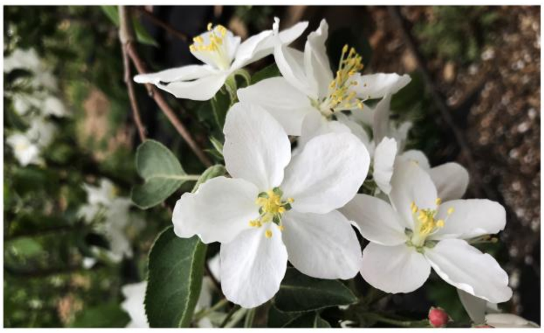

M. sieversii subsp. xinjinensis, Type Ⅱ [1 1 1 1 1 0]

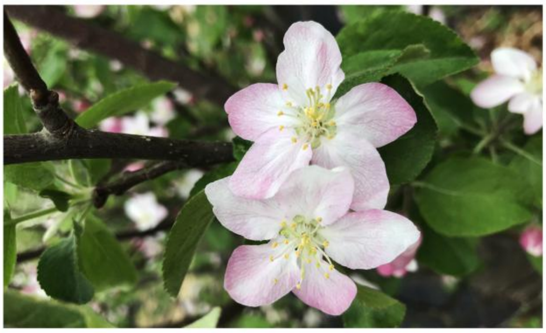

M. sylvestris, Type Ⅱ [1 1 1 1 1 0]

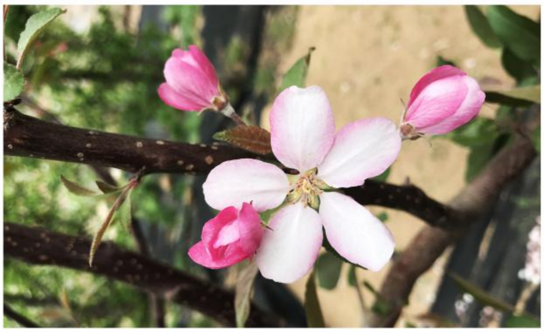

M. fusca, Type Ⅲ [1 1 1 0 1 1]

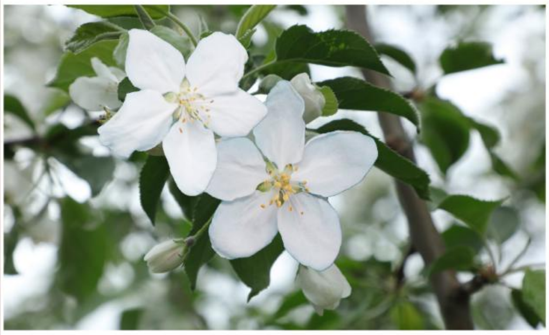

M. domestica var. *binzi*, Type Ⅳ [1 1 1 0 1 0]

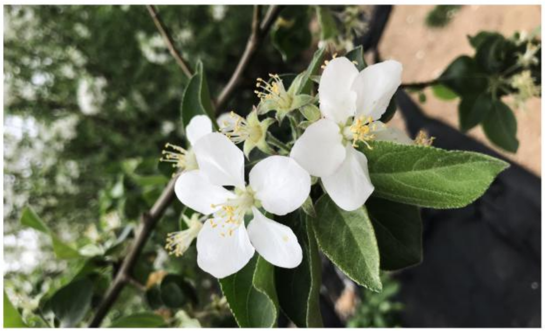

M. kirghisorum, Type Ⅳ [1 1 1 0 1 0]

Species

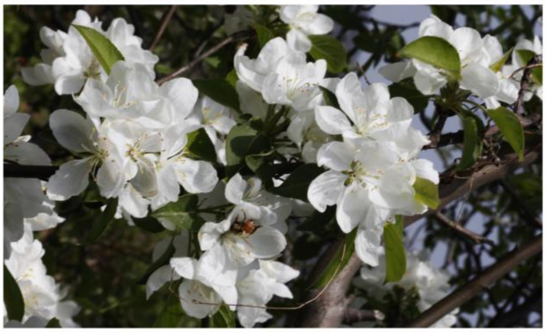

M. prunifolia, Type Ⅳ[1 1 1 0 0 0]

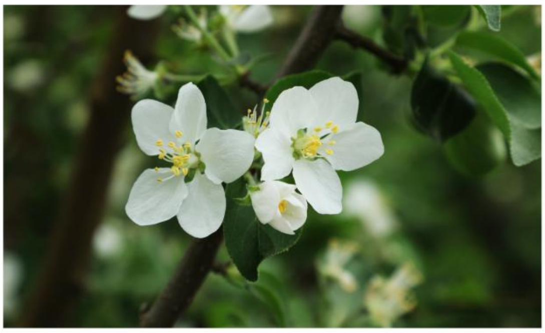

M. rockii, Type Ⅳ[1 1 1 0 1 0]

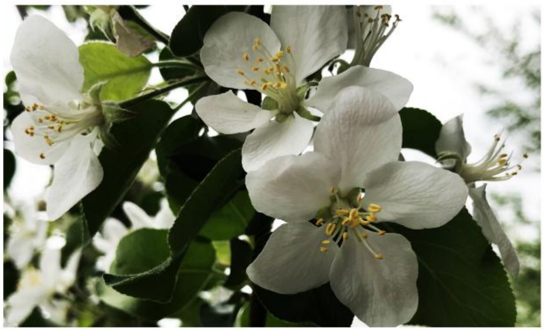

M. sieversii, Type Ⅳ[1 1 1 0 1 0]

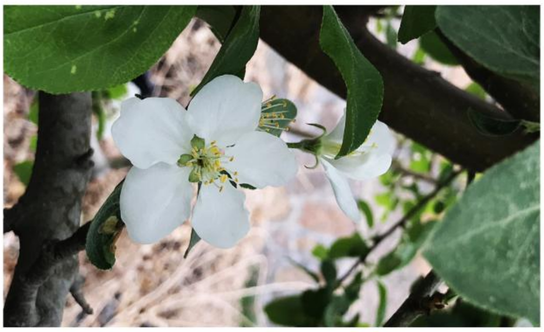

M. turkmenorum, Type Ⅳ[1 1 1 0 1 0]

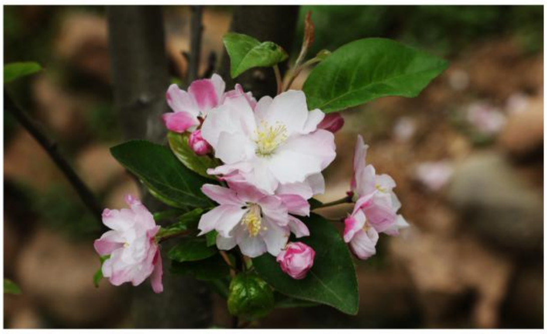

M. spectabilis, Type Ⅶ[0 1 0 0 1 0]

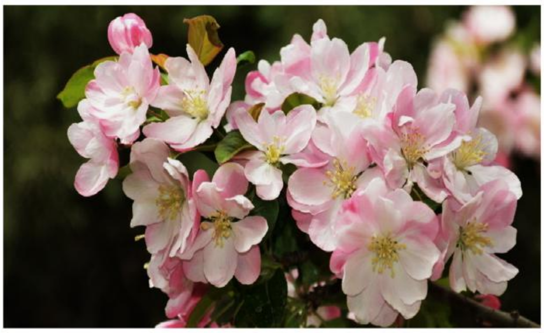

M. micromalus, Type Ⅶ[0 1 0 0 1 0]

Cultivars

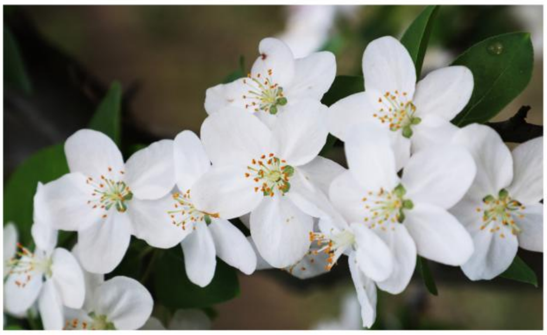

M. 'Butterball', Type Ⅰ [1 1 1 1 1 1]

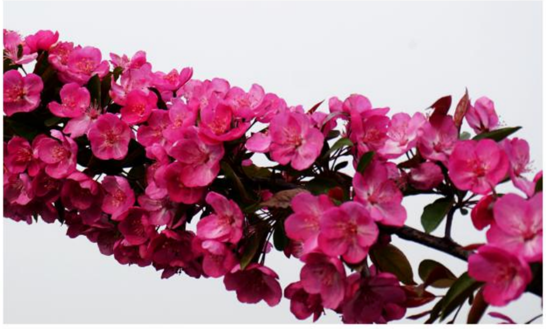

M. 'Cardinal', Type Ⅰ [1 1 1 1 1 1]

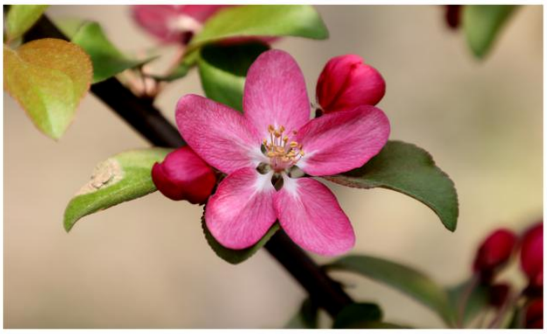

M. 'Centurion', Type Ⅰ [1 1 1 1 1 1]

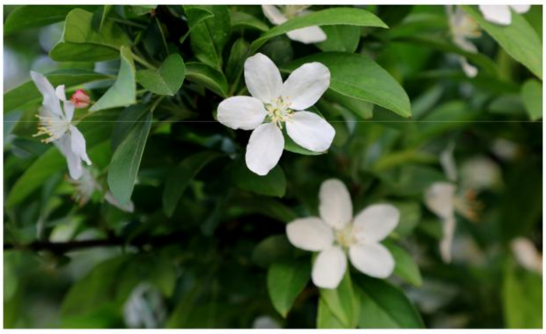

M. 'Cinderella', Type Ⅰ [1 1 1 1 1 1]

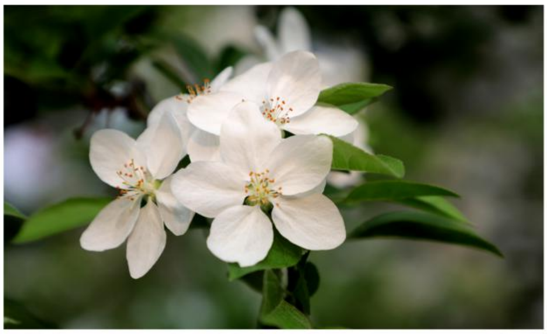

M. 'Guard', Type Ⅰ [1 1 1 1 1 1]

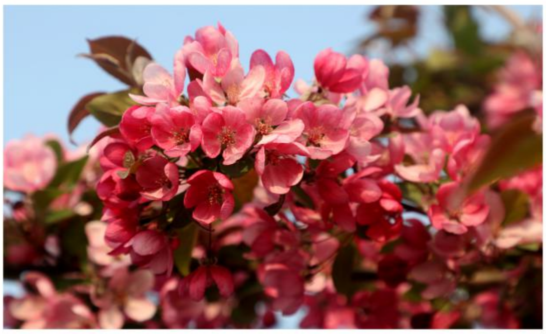

M. 'Indian Magic', Type Ⅰ [1 1 1 1 1 1]

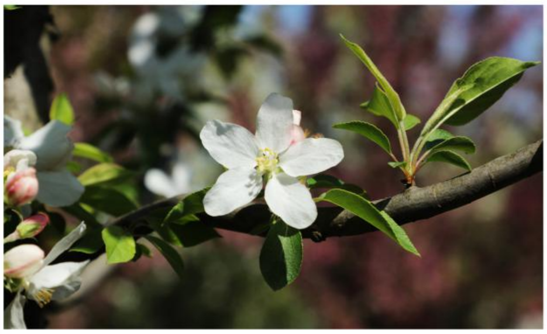

M. 'Irene', Type Ⅰ [1 1 1 1 1 1]

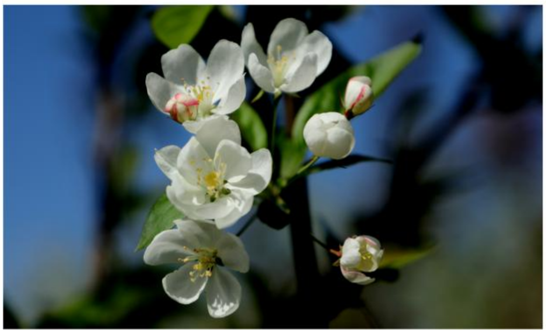

M. 'Melaleuca Bracteata', Type Ⅰ [1 1 1 1 1 1]

Cultivars

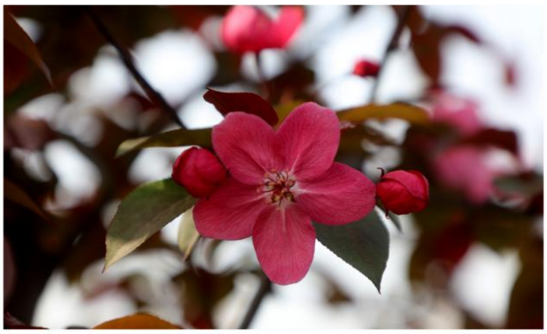

M. ‘Perfect Purple’, Type Ⅰ[1 1 1 1 1 1]

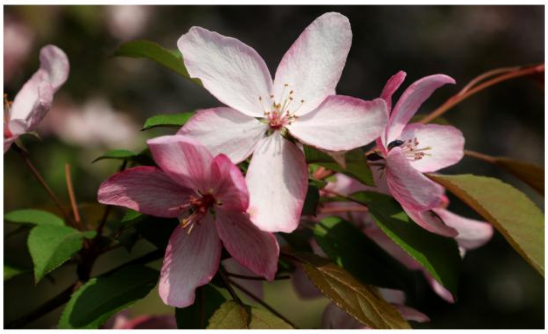

M. ‘Pink Spires’, Type Ⅰ[1 1 1 1 1 1]

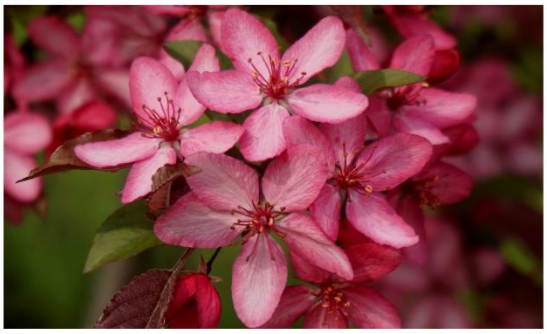

M. ‘Royal Raindrop’, Type Ⅰ[1 1 1 1 1 1]

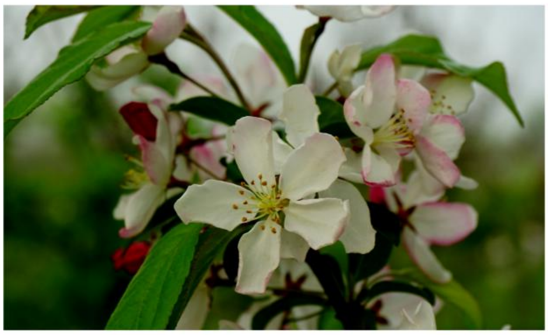

M. ‘Sentinel’, Type Ⅰ[1 1 1 1 1 1]

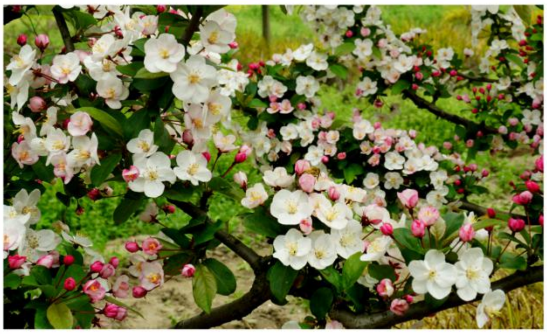

M. ‘Spring Sensation’, Type Ⅰ[1 1 1 1 1 1]

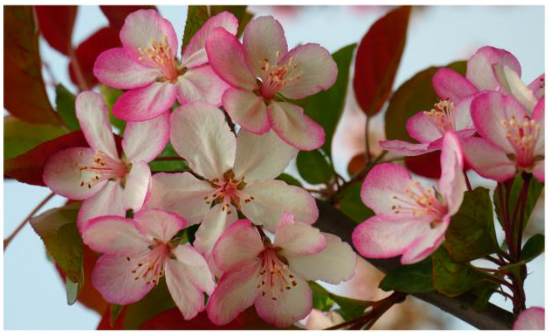

M. ‘Strawberry Jelly’, Type Ⅰ[1 1 1 1 1 1]

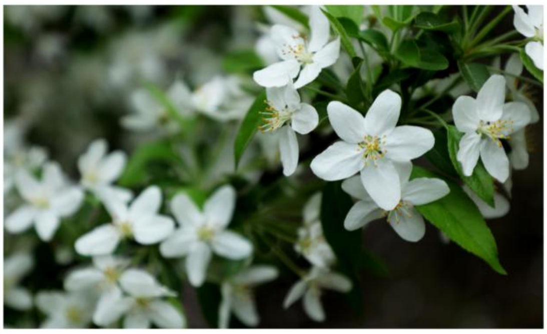

M. ‘Sugar Tyme’, Type Ⅰ[1 1 1 1 1 1]

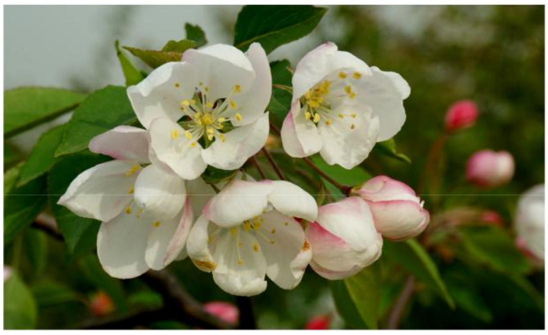

M. ‘Weeping Madonna’, Type Ⅰ[1 1 1 1 1 1]

Cultivars

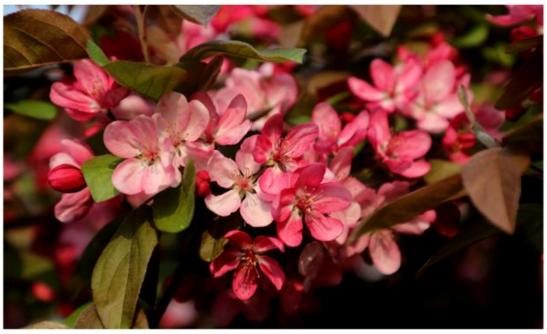

M. 'Adams', Type Ⅱ [1 1 1 1 1 0]

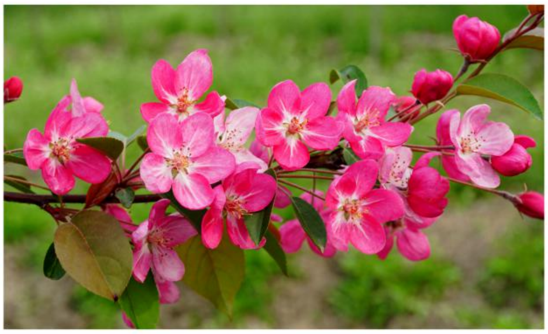

M. 'Black Jade', Type Ⅱ [1 1 1 1 0 0]

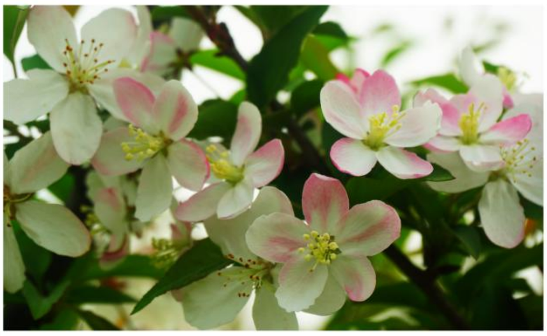

M. 'Bride', Type Ⅱ [1 1 1 1 1 0]

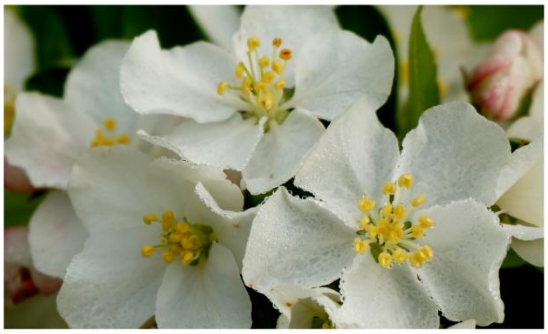

M. 'Lollipop', Type Ⅱ [1 1 1 1 0 0]

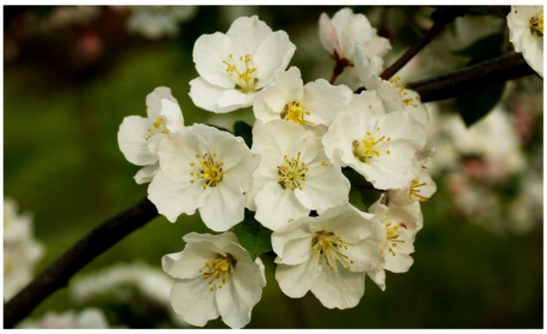

M. 'Mary Potter', Type Ⅱ [1 1 1 1 1 0]

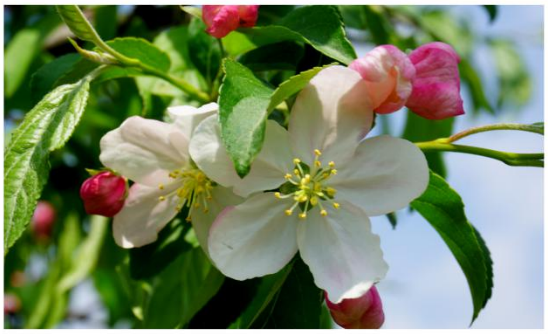

M. 'Molten Lava', Type Ⅱ [1 1 1 1 1 0]

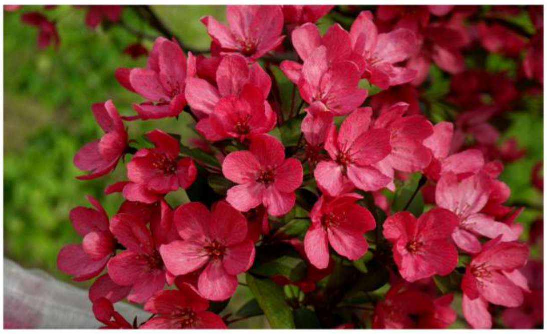

M. 'Prairifire', Type Ⅱ [1 1 1 1 1 0]

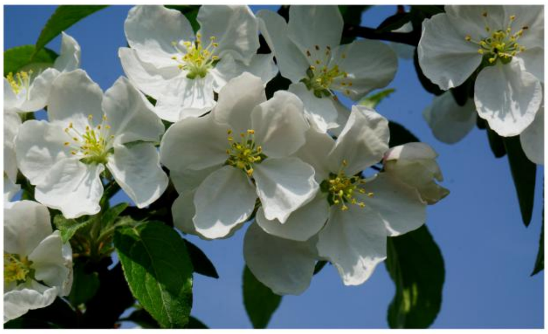

M. 'Professor Sprenger', Type Ⅱ [1 1 1 1 1 0]

Cultivars

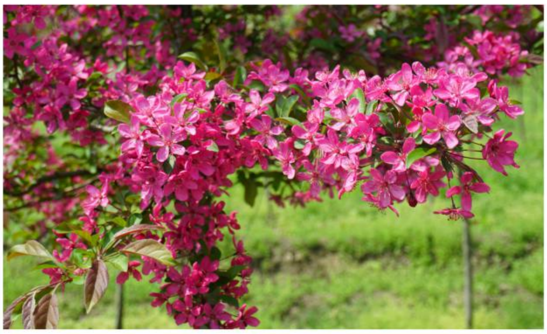

M. 'Purple Prince', Type Ⅱ [1 1 1 1 1 0]

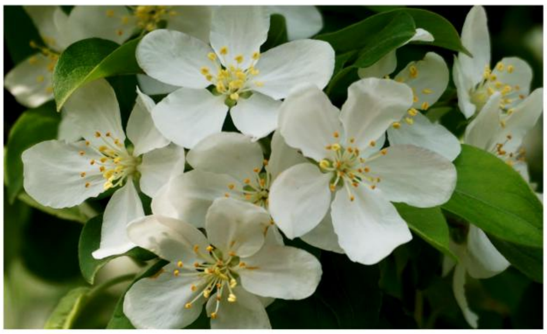

M. 'Rainbow', Type Ⅱ [1 1 1 1 1 0]

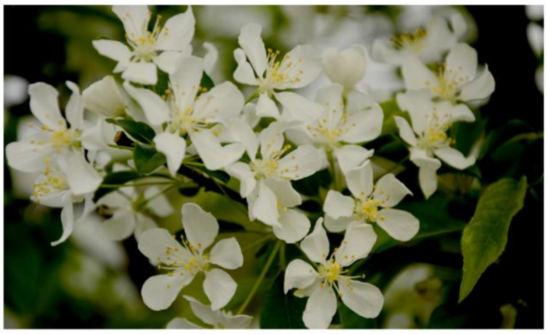

M. 'Red Jewel', Type Ⅱ [1 1 1 1 0 0]

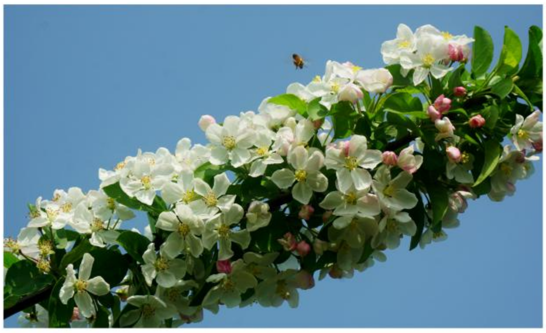

M. 'Red Sentinel', Type Ⅱ [1 1 1 1 0 0]

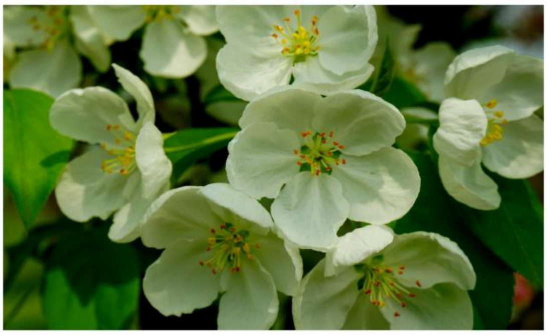

M. 'Snowdrift', Type Ⅱ [1 1 1 1 1 0]

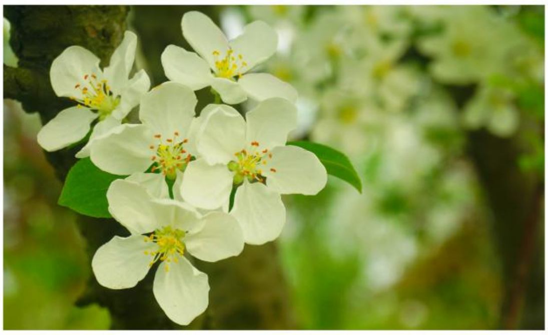

M. 'Winter Red', Type Ⅱ [1 1 1 1 1 0]

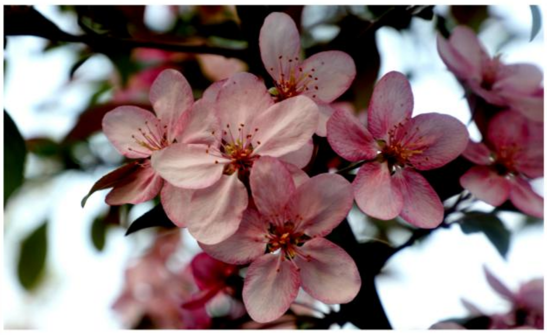

M. 'Coccinella', Type Ⅲ [1 1 1 0 1 1]

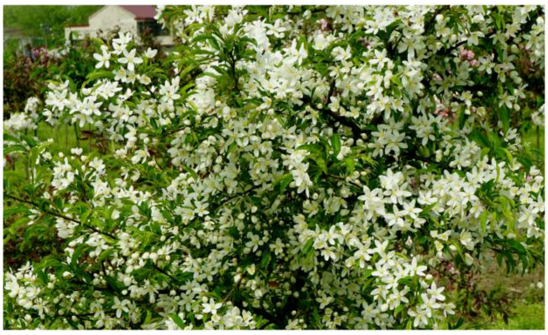

M. 'Golden Raindrop', Type Ⅲ [1 1 1 0 1 1]

Cultivars

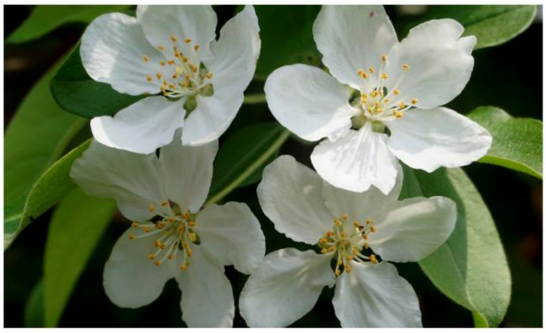

M. ‘Gorgeous’, Type Ⅲ [1 1 1 0 1 1]

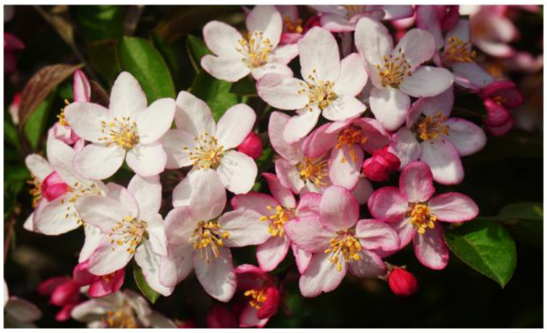

M. ‘May's Delight’, Type Ⅲ [1 1 0 0 1 1]

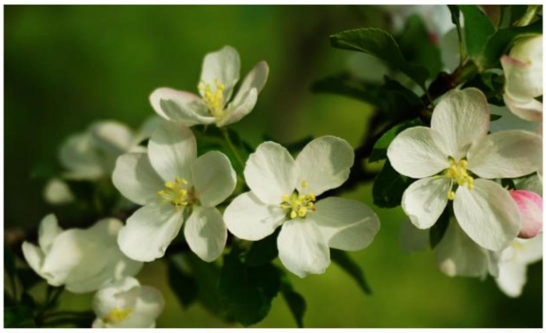

M. ‘Shelley’, Type Ⅲ [1 1 0 0 1 1]

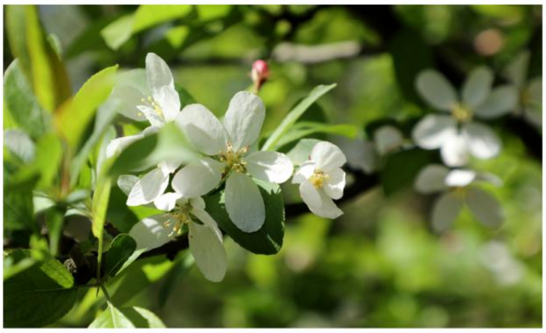

M. ‘Winter Gold’, Type Ⅲ [1 1 1 0 1 1]

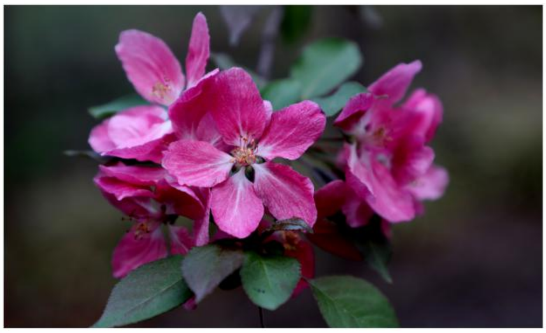

M. ‘Abundance’, Type Ⅳ [1 1 1 0 1 0]

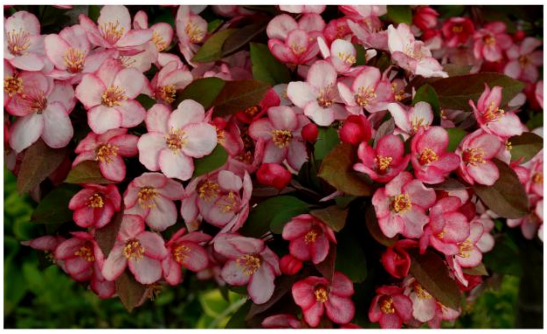

M. ‘Candymint’, Type Ⅳ [1 1 0 0 1 0]

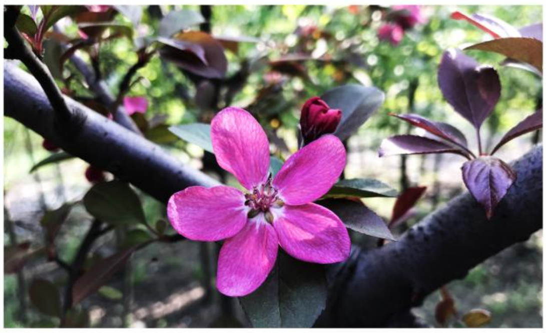

M. ‘Darwin’, Type Ⅳ [1 1 1 0 1 0]

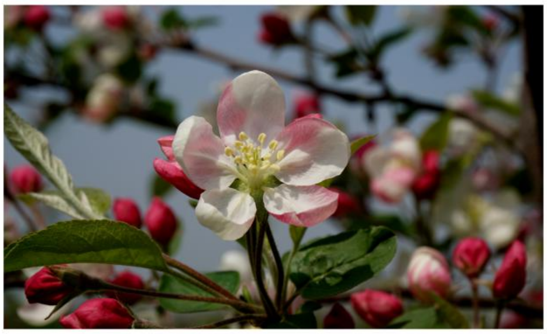

M. ‘Everest’, Type Ⅳ [1 1 0 0 1 0]

Cultivars

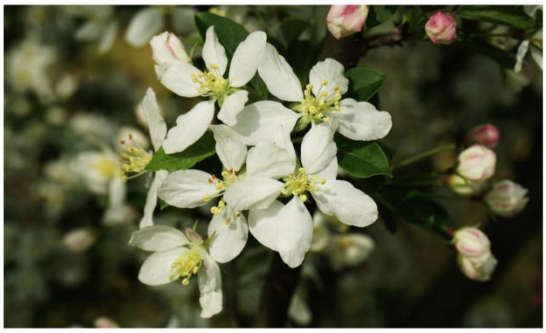

M. 'Fairytail Gold', Type Ⅳ[1 1 1 0 1 0]

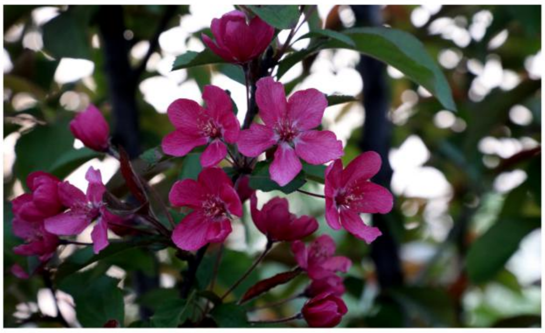

M. 'Flame', Type Ⅳ[1 1 0 0 0 0]

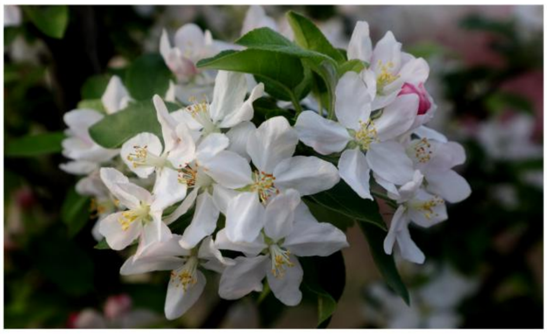

M. 'Golden Hornet', Type Ⅳ[1 1 0 0 0 0]

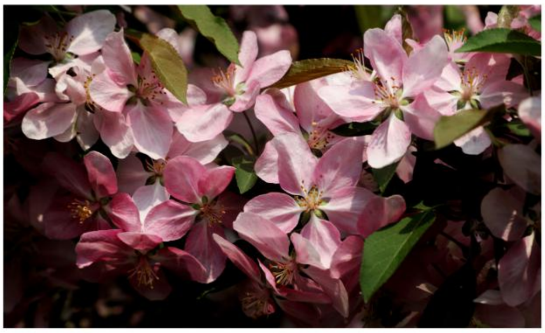

M. 'Hopa', Type Ⅳ[1 1 1 0 1 0]

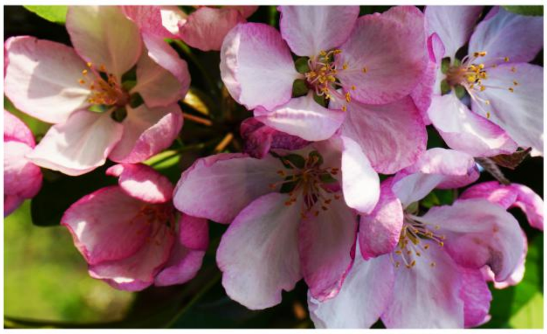

M. 'Indian Summer', Type Ⅳ[1 1 1 0 0 0]

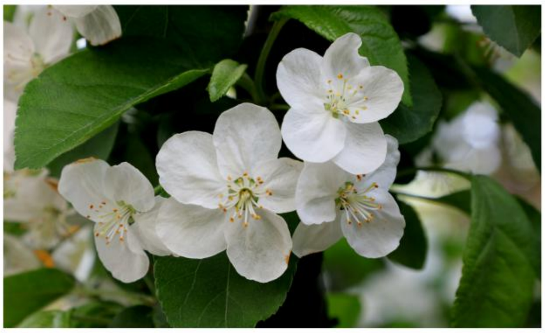

M. 'King Arthur', Type Ⅳ[1 1 1 0 1 0]

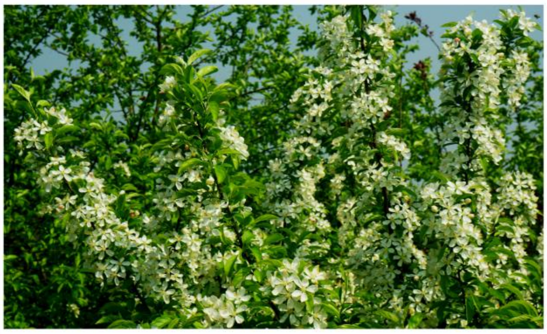

M. 'Lancelot', Type Ⅳ[1 1 0 0 0 0]

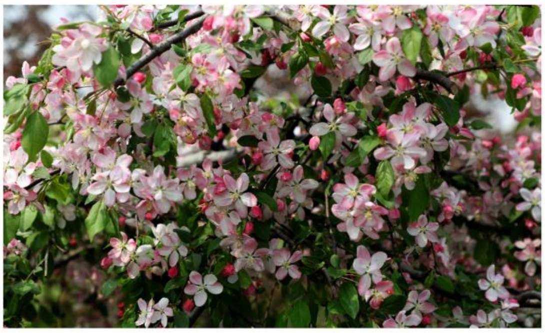

M. 'Louisa', Type Ⅳ[1 1 1 0 1 0]

Cultivars

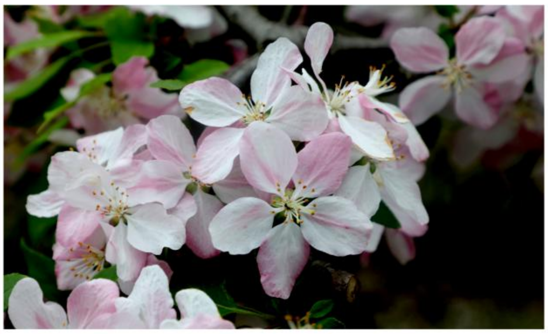

M. ‘Louisa Contort’, Type Ⅳ[1 1 1 0 1 0]

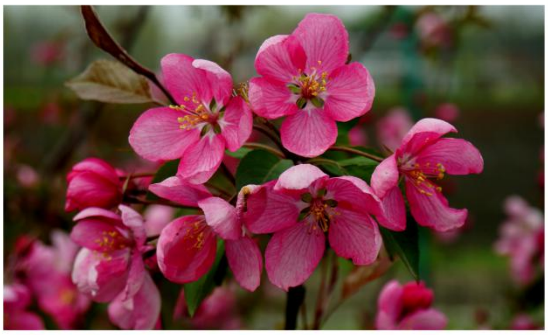

M. ‘Makamik’, Type Ⅳ[1 1 1 0 0 1]

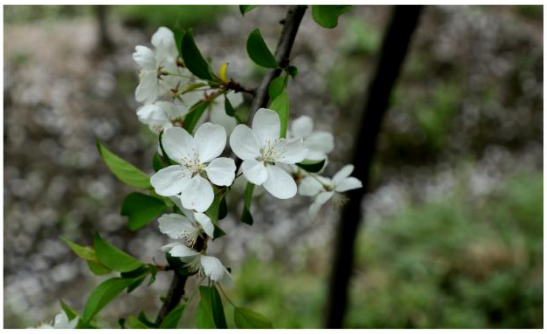

M. ‘Orange Dream’, Type Ⅳ[1 1 1 0 0 0]

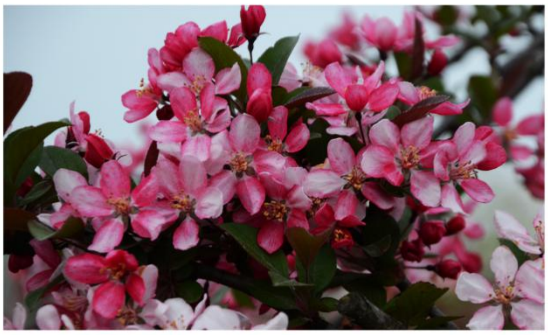

M. ‘Pink Princess’, Type Ⅳ[1 1 1 0 1 0]

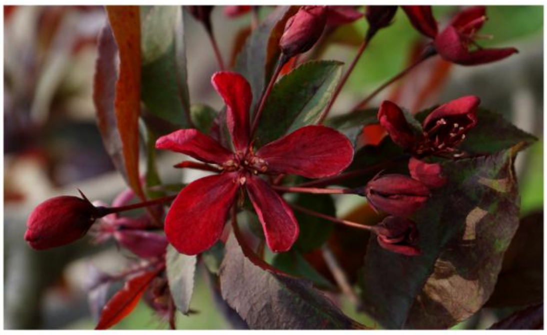

M. ‘Purple Gem’, Type Ⅳ[1 1 1 0 1 0]

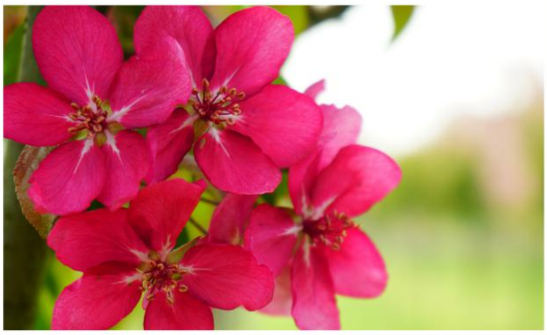

M. ‘Red Baron’, Type Ⅳ[1 1 1 0 0 0]

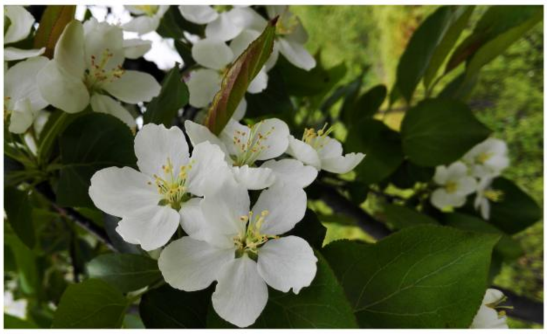

M. ‘Red Coral’, Type Ⅳ[1 1 1 0 0 0]

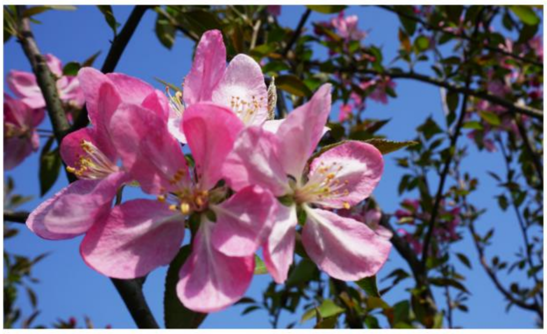

M. ‘Red Great’, Type Ⅳ[1 1 1 0 1 0]

Cultivars

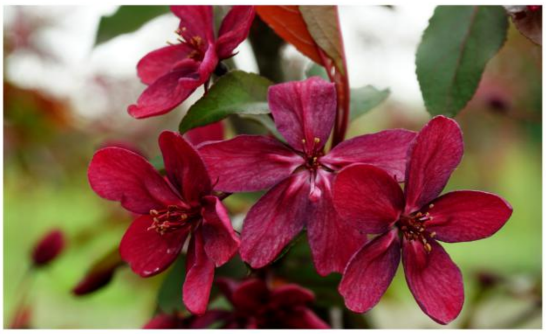

M. 'Royalty', Type Ⅳ[1 1 1 0 1 0]

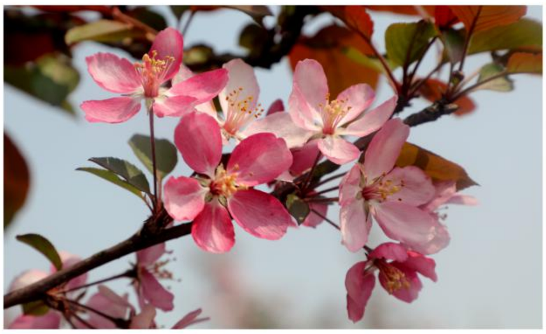

M. 'Thunder child', Type Ⅳ[1 1 1 0 0 1]

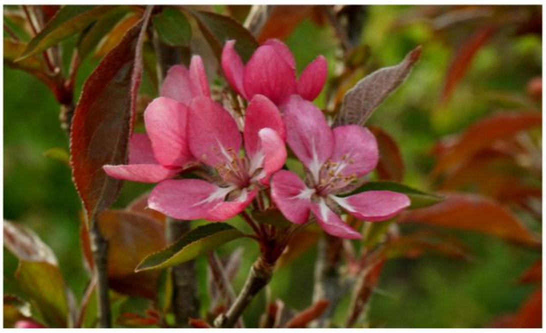

M. 'Velvet Pillar', Type Ⅳ[1 1 1 0 0 0]

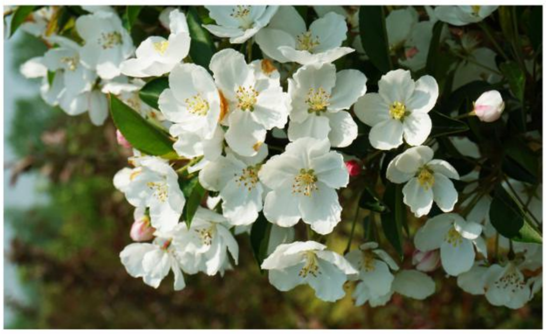

M. 'Adirondack', Type Ⅴ[1 0 1 1 0 0]

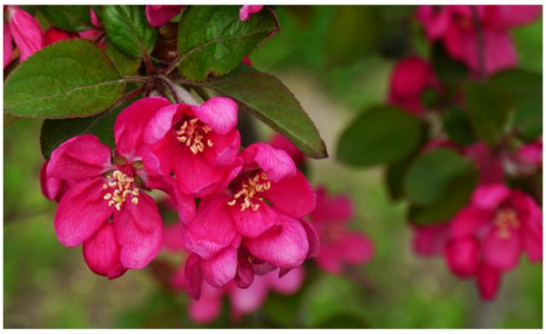

M. 'John Downie', Type Ⅴ[1 0 1 1 0 0]

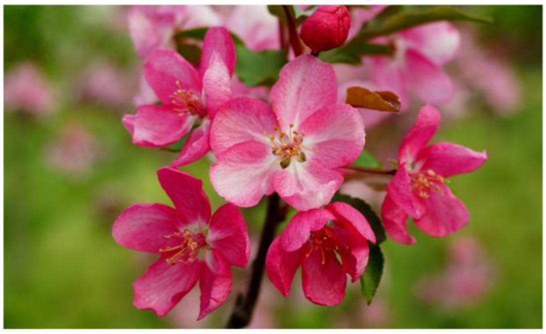

M. 'Rudolph', Type Ⅴ[1 0 1 1 0 0]

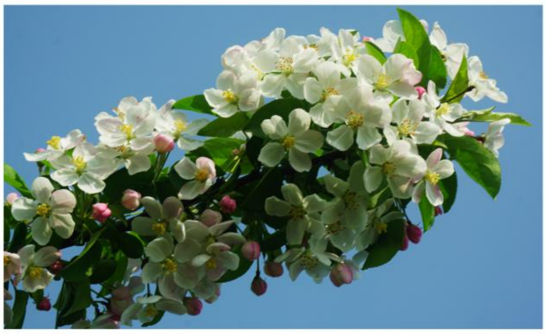

M. 'Sweet Sugar tyme', Type Ⅴ[0 1 1 1 1 0]

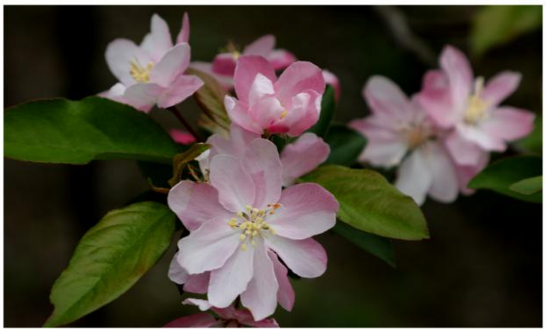

M. 'Ballet', Type Ⅵ[0 1 0 0 1 1]

Cultivars

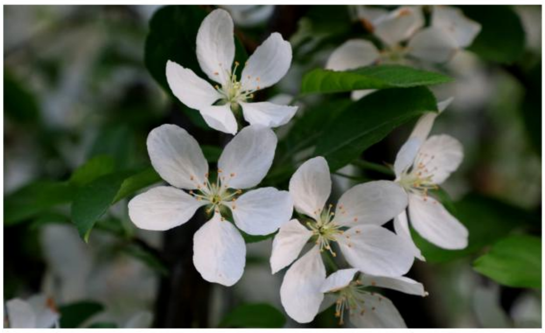

M. 'Harvest Gold', Type Ⅵ [0 1 1 0 1 1]

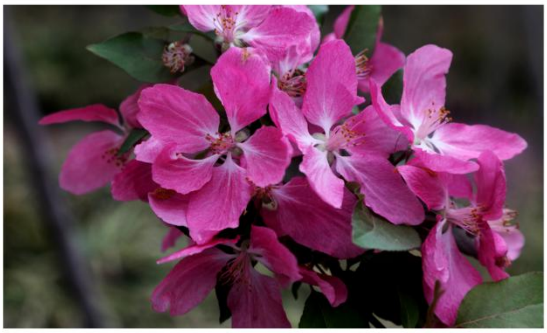

M. 'Almey', Type Ⅶ [1 0 1 0 0 0]

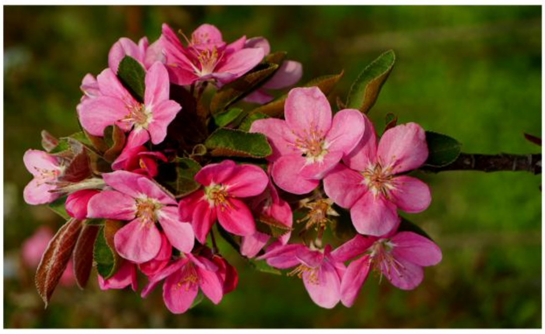

M. 'Ballet Red', Type Ⅶ [1 0 1 0 0 0]

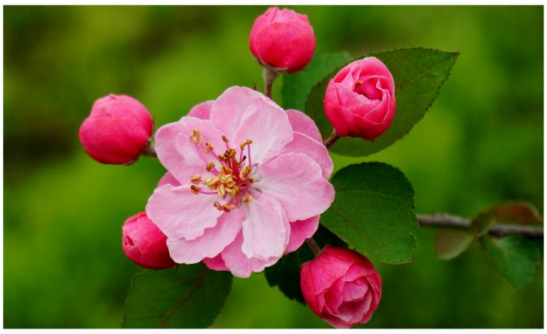

M. 'Brandywine', Type Ⅶ [0 1 1 0 1 0]

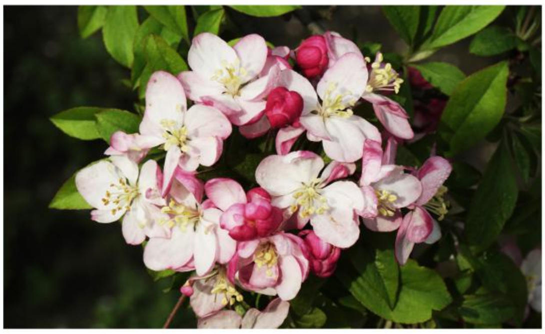

M. 'Coralburst', Type Ⅶ [0 0 0 0 0 0]

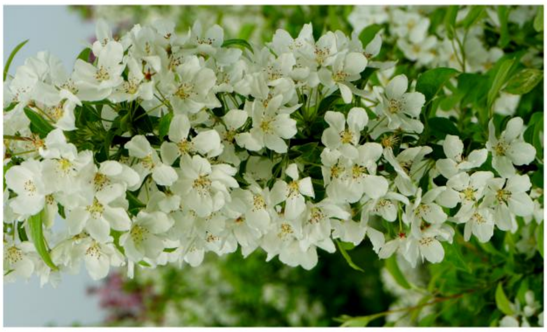

M. 'David', Type Ⅶ [1 0 1 0 0 0]

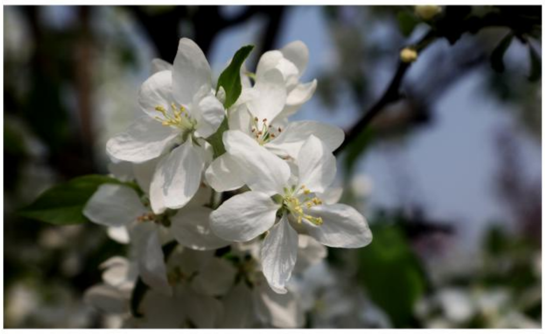

M. 'Dolgo', Type Ⅶ [1 0 1 0 0 1]

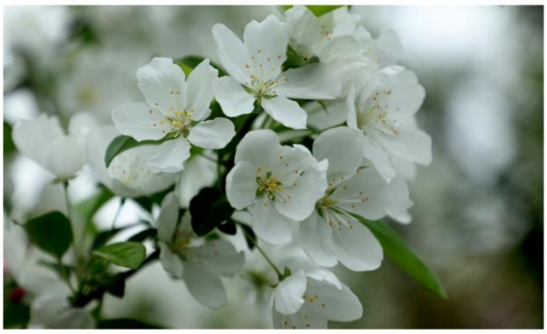

M. 'Donald Wyman', Type Ⅶ [0 0 1 0 0 0]

Cultivars

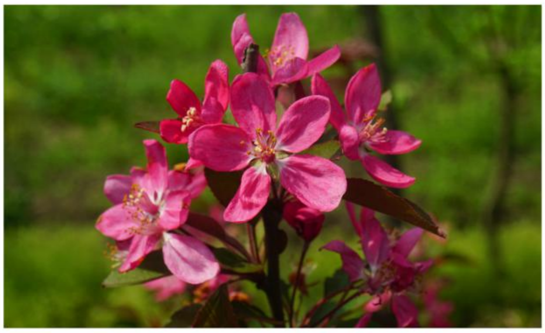

M. 'Eleyi', Type Ⅶ [1 0 0 0 0 0]

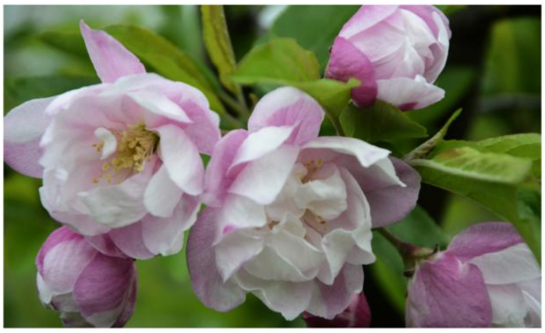

M. 'Fen Balei', Type Ⅶ [0 0 0 0 0 0]

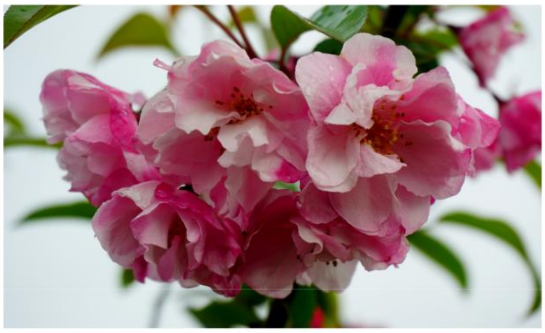

M. 'Fenhong nichang', Type Ⅶ [0 0 0 0 0 0]

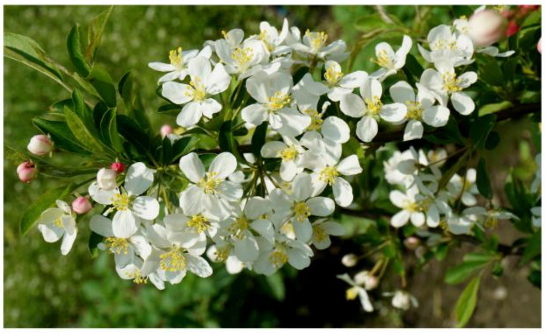

M. 'Firebird', Type Ⅶ [0 1 0 0 0 0]

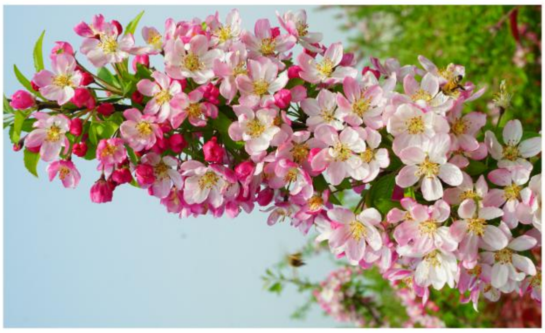

M. 'Hillier', Type Ⅶ [0 0 0 0 0 0]

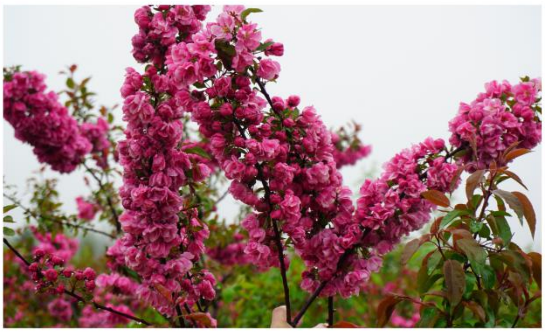

M. 'Kelsey', Type Ⅶ [0 0 0 0 0 0]

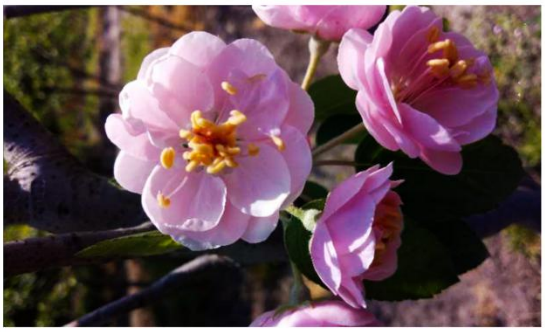

M. 'Klehm's Improved Bechtel', Type Ⅶ [0 0 0 0 0 0]

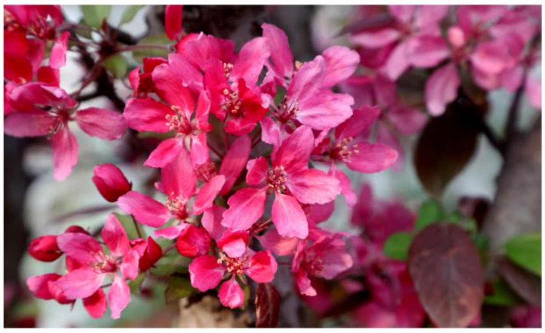

M. 'Lisa', Type Ⅶ [1 0 0 0 0 0]

Cultivars

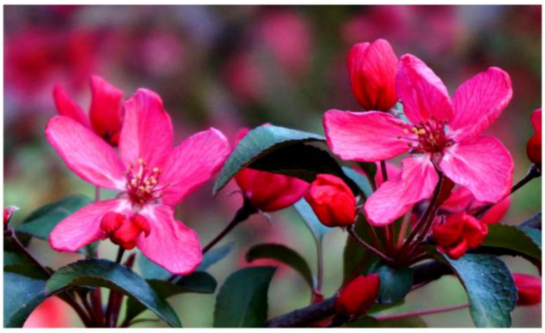

M. 'Liset', Type Ⅶ [1 0 1 0 0 0]

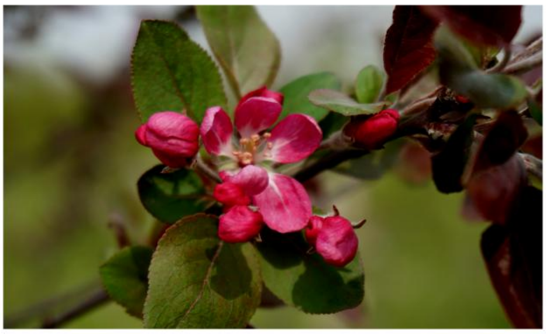

M. 'Neville Copeman', Type Ⅶ [0 0 0 0 0 0]

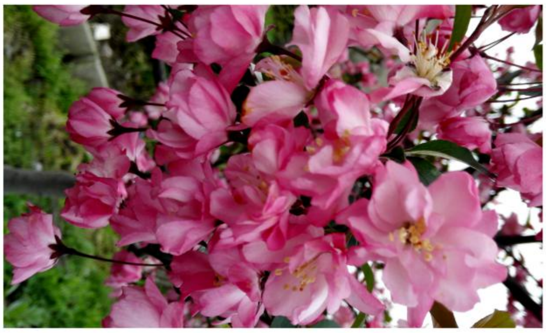

M. 'Pink Double', Type Ⅶ [0 0 0 0 0 0]

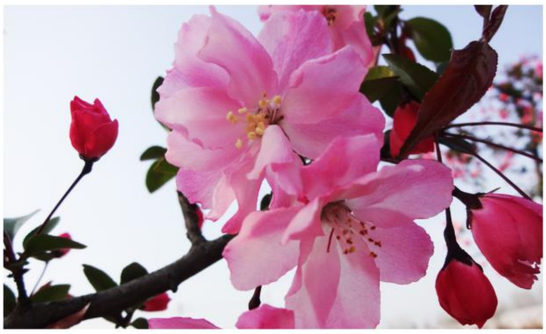

M. 'Pink Double NFU', Type Ⅶ [0 0 0 0 0 0]

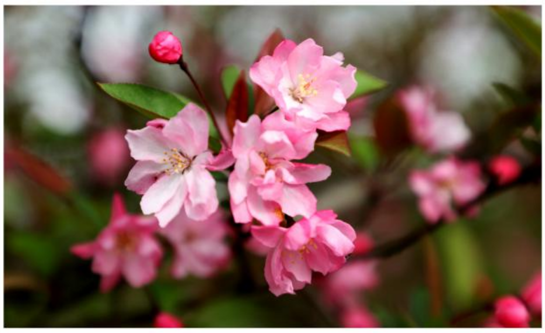

M. 'Pink Pillar', Type Ⅶ [0 0 0 0 0 0]

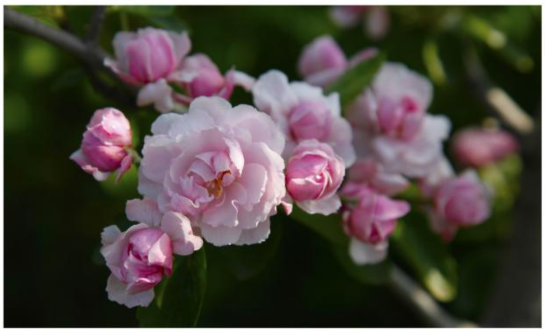

M. 'Praire Rose', Type Ⅶ [0 0 0 0 0 0]

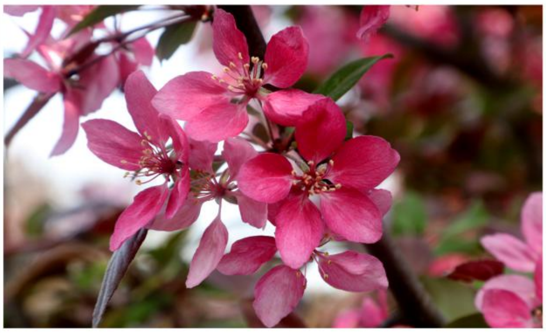

M. 'Profusion', Type Ⅶ [0 0 1 0 0 0]

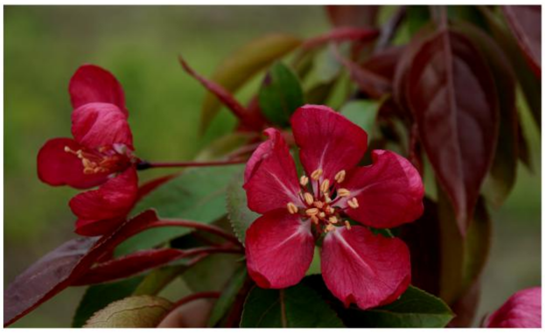

M. 'Purple Pendula', Type Ⅶ [1 0 1 0 0 0]

Cultivars

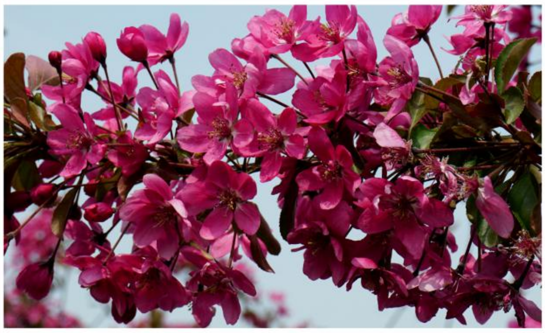

M. 'Purple Spring', Type Ⅶ[1 0 1 0 0 0]

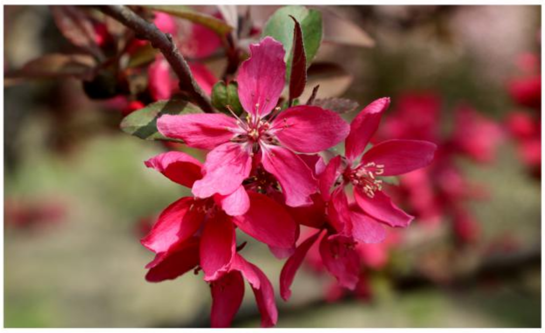

M. ×purpurea'Lemoinei', Type Ⅶ[1 0 1 0 0 0]

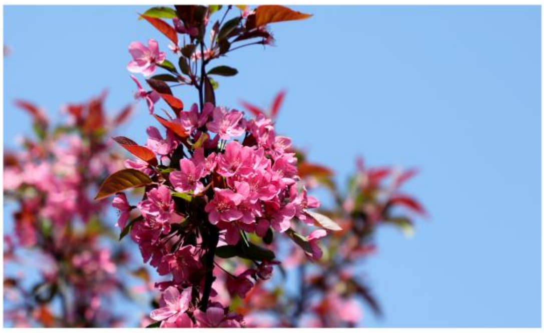

M. 'Radiant', Type Ⅶ[1 0 1 0 0 0]

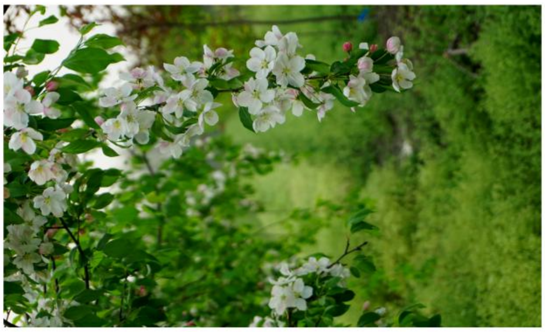

M. 'Red Jade', Type Ⅶ[1 0 1 0 0 0]

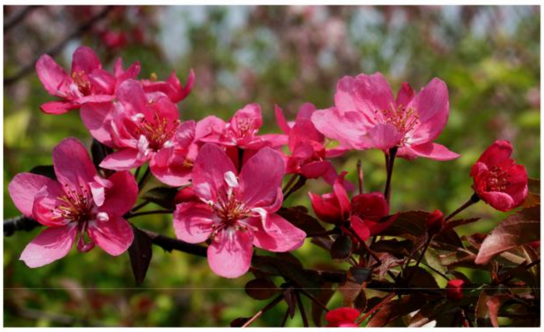

M. 'Red Nessy', Type Ⅶ[1 0 0 0 0 0]

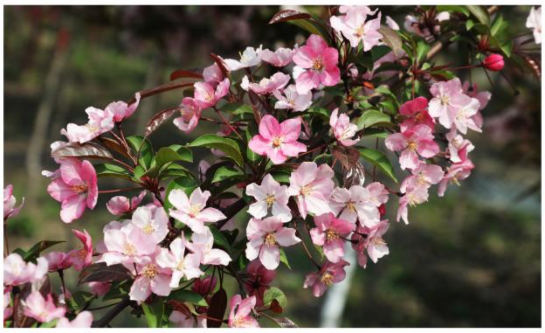

M. 'Red Splendor', Type Ⅶ[1 0 1 0 0 0]

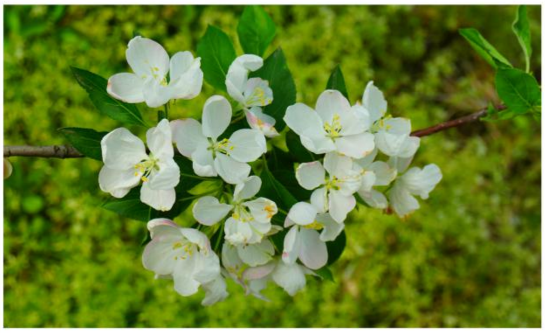

M. 'Regal', Type Ⅶ[0 0 0 0 0 0]

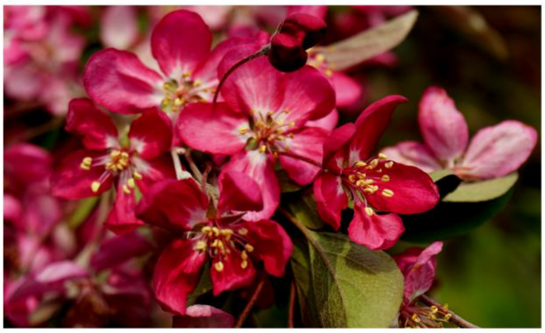

M. 'Robinson', Type Ⅶ[1 0 0 0 0 0]

Cultivars

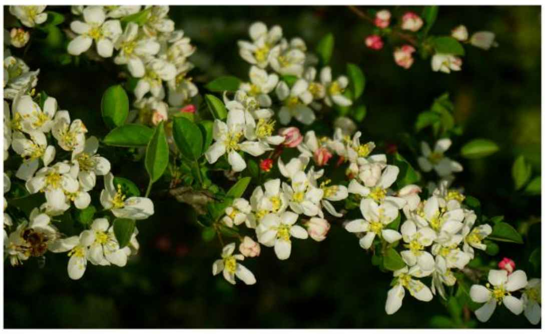

M. 'Roger's Selection', Type Ⅶ [0 0 1 0 0 0]

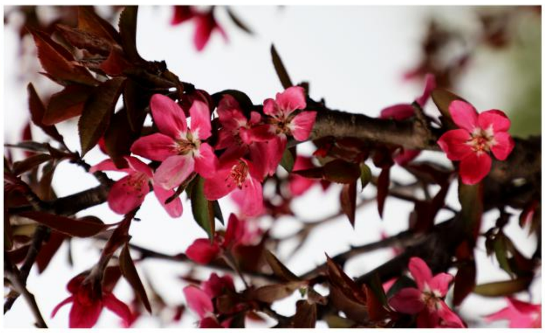

M. 'Royal Gem', Type Ⅶ [0 0 1 0 0 0]

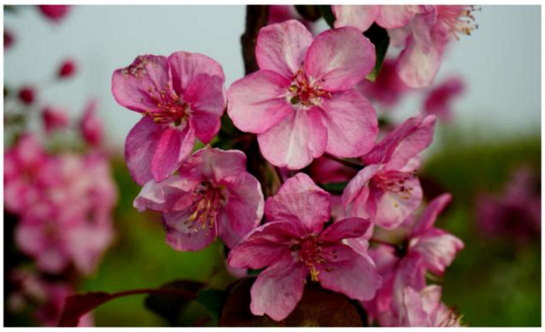

M. 'Selkirk', Type Ⅶ [1 0 1 0 0 0]

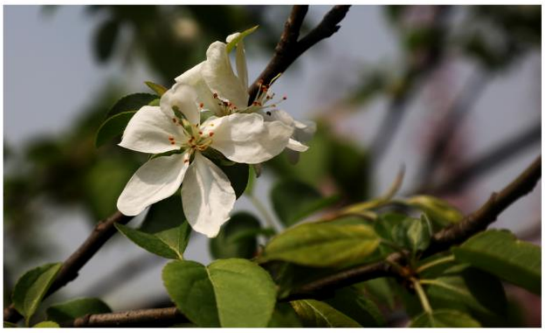

M. 'Show Girl', Type Ⅶ [0 0 0 0 0 0]

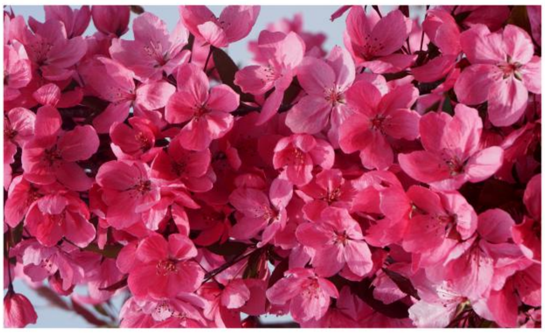

M. 'Show Time', Type Ⅶ [1 0 0 0 0 0]

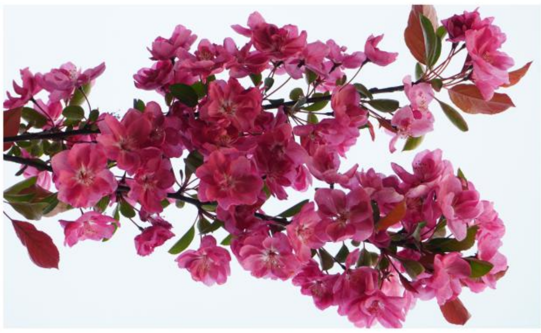

M. 'Sparkler', Type Ⅶ [0 0 0 0 0 0]

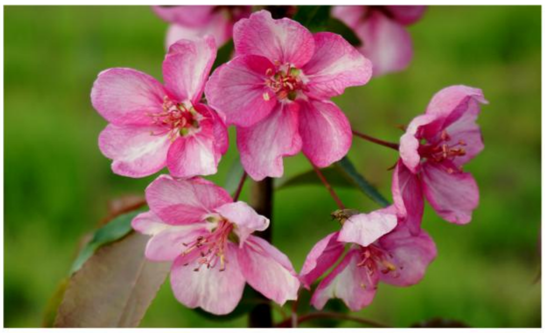

M. 'Spring Glory', Type Ⅶ [1 0 1 0 0 0]

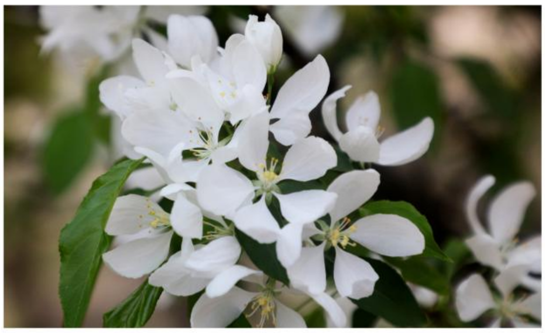

M. 'Spring Snow', Type Ⅶ [1 0 1 0 0 1]

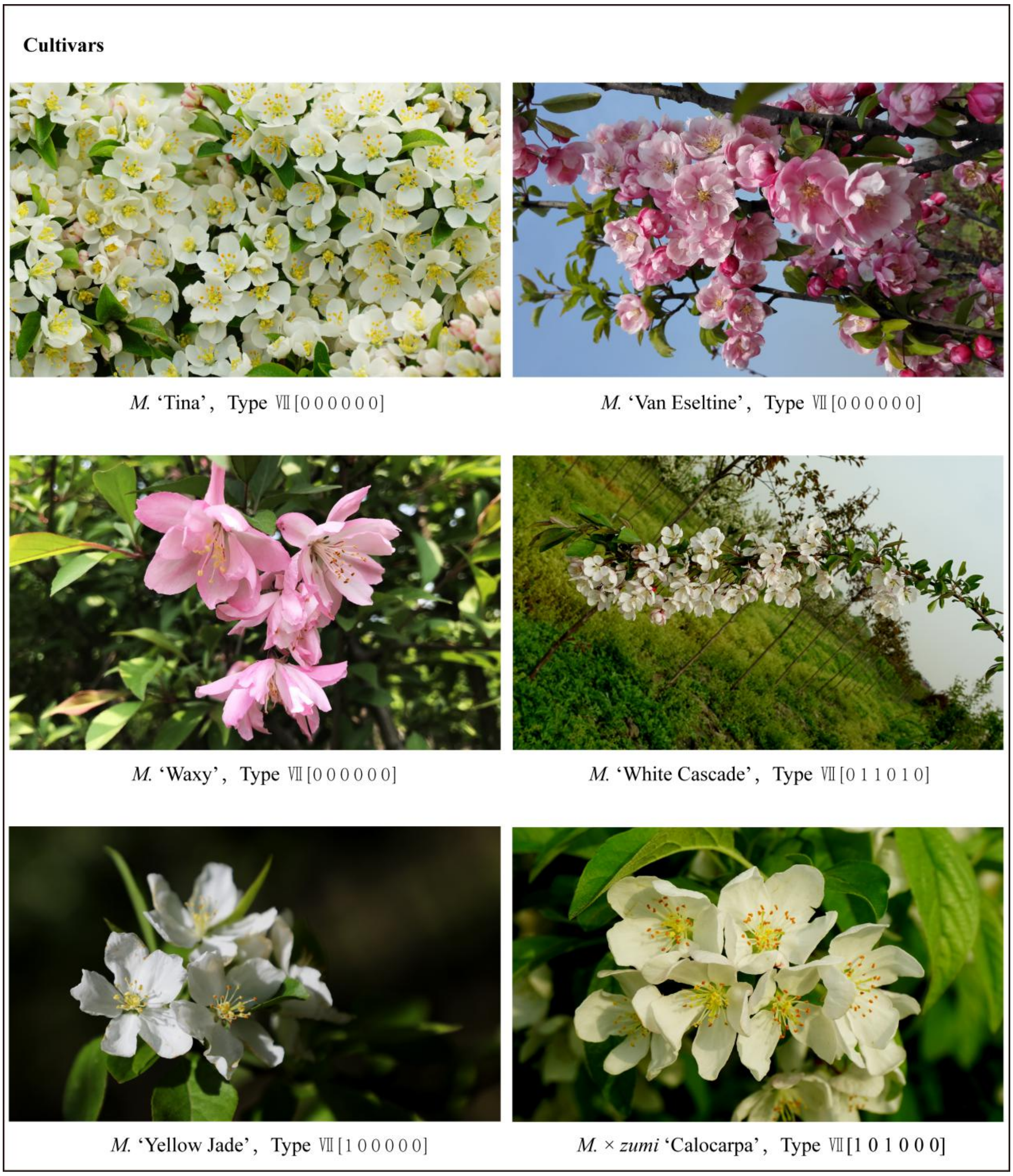

Figure S1. Corolla symmetry images of all 140 *Malus* taxa. The matrix form of symmetry is original which contains six sub-dimensions (petal interval, X_1; petal coplanarity, X_2; petal shape homogeneity, Y_1; petal size homogeneity, Y_2; petal local curling consistency, Z_1; petal local wrinkle consistency, Z_2).
